# Irregular G-quadruplexes Found in the Untranslated Regions of Human mRNAs Influence Translation*[Fn FN2]

**DOI:** 10.1074/jbc.M116.744839

**Published:** 2016-08-24

**Authors:** François Bolduc, Jean-Michel Garant, Félix Allard, Jean-Pierre Perreault

**Affiliations:** From the RNA Group/Groupe ARN, Département de Biochimie, Faculté de Médecine et Sciences de la Santé, Pavillon de Recherche Appliquée au Cancer, Université de Sherbrooke, Sherbrooke, Quebec J1E 4K8, Canada

**Keywords:** G-quadruplex, gene expression, RNA, RNA structure, translation, in-line probing

## Abstract

G-quadruplex structures are composed of coplanar guanines and are found in both DNA and RNA. They are formed by the stacking of two or more G-quartets that are linked together by three loops. The current belief is that RNA G-quadruplexes include loops of l to 7 nucleotides in length, although recent evidence indicates that the central loop (loop 2) can be longer if loops 1 and 3 are limited to a single nucleotide each. With the objective of broadening the definition of irregular RNA G-quadruplexes, a bioinformatic search was performed to find potential G-quadruplexes located in the untranslated regions of human mRNAs (*i.e.* in the 5′ and 3′-UTRs) that contain either a long loop 1 or 3 of up to 40 nucleotides in length. RNA molecules including the potential sequences were then synthesized and examined *in vitro* by in-line probing for the formation of G-quadruplex structures. The sequences that adopted a G-quadruplex structure were cloned into a luciferase dual vector and examined for their ability to modulate translation *in cellulo*. Some irregular G-quadruplexes were observed to either promote or repress translation regardless of the position or the size of the long loop they possessed. Even if the composition of a RNA G-quadruplex is not quite completely understood, the results presented in this report clearly demonstrate that what defines a RNA G-quadruplex is much broader than what we previously believed.

## Introduction

G-quadruplexes (G4)^4^ are secondary structures involving four nucleic acid strands that can be adopted by both DNA and RNA that contains guanine-rich sequences. These structures rely on the formation of Hoogsteen base pairs that generate a quartet of guanine residues. The parallel and continuous stacking of at least two such quartets, which is stabilized by the presence of a monovalent cation (K^+^ > Na^+^ ≫ Li^+^), constitutes the G4. Intramolecular G4-forming sequences are usually described by four stretches of consecutive guanine residues that are separated by short loops, that is to say sequences such as G_3_N*_x_*G_3_N*_x_*G_3_N*_x_*G_3_ in which N can be any nucleotide, and *x* is usually 7 or fewer (1, 2).

DNA G-quartets and G4 were characterized for the first time in 1962 using x-ray diffraction (3). They have been studied in great detail *in vitro*, and it has been shown that they are formed under physiological conditions of both salinity and pH (4). Moreover, numerous proteins are capable of binding to G4 motifs and then either stabilizing or unwinding them (5). The presence of DNA G4 structures has been recently visualized in a cell (6). These DNA structures have been shown to be implicated in both transcriptional regulation and telomere structure (7, 8). RNA G4, which are thermodynamically more stable than DNA G4 (9–12), have also been visualized in human cells (13), and numerous regulatory functions have been attributed to them. Predominantly found in the untranslated regions (UTR) of mRNA and introns, they have been shown to play roles in splicing (14, 15), polyadenylation (16), translational regulation (17–21), cellular localization of mRNA (22), and telomere length maintenance (23). All of these regulatory functions make them very appealing as therapeutic targets (24).

**FIGURE 1.**
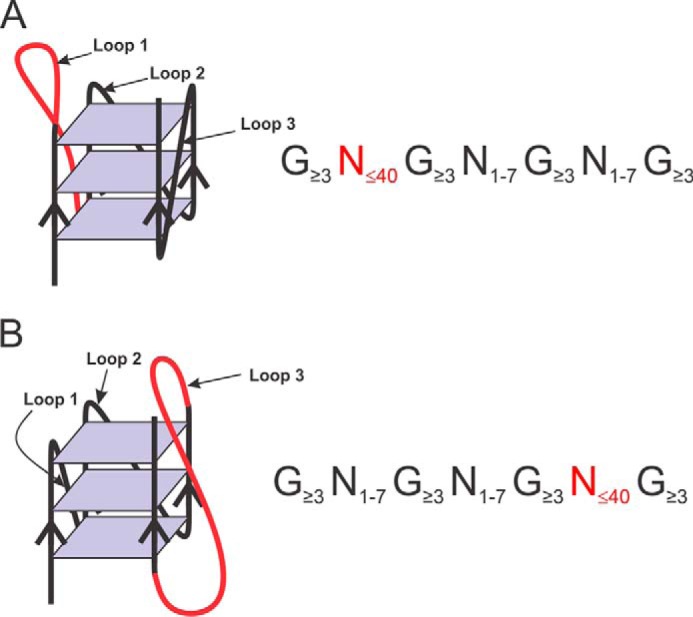
**Schematic representation of long loop 1 (*A*) and long loop 3 (*B*) RNA G-quadruplexes.** The long loops are represented by the *red lines*. Beside each G4, the expression used for the python script is shown.

The topology, that is to say the composition and the loop lengths of RNA G4s, has been the subject of a few studies (12, 25, 26). In all cases it was demonstrated that G4 stability is inversely related to loop length. Moreover, the sizes of the loops were always between one and seven nucleotides (nt), with three exceptions. The first of these was a biophysical study that permitted the characterization of G4 structures that possessed a central loop (loop 2; see Fig. 1) of up to 15 nt in length (26). The second was the investigation of naturally occurring G4 found in human 5′-UTRs that possessed a relatively long loop 2 of 8–70 nt (20). Although G4 sequences that included a loop 2 of up to 70 nt were shown to fold correctly in solution, the repression of translation *in cellulo* was only observed with G4 motifs possessing loops of up to 32 nt in length. The third exception was the report of two naturally occurring G4 in human mRNA that possessed long loop 2 of 12 and 13 nt and where the formation of the G4 structure could be modulated using small antisense oligonucleotides (17). It is noteworthy that all of these studies involved G4 motifs with oversized loop 2 and loops 1 and 3 composed solely of 1 nt. Clearly, the classical definition of a G4 (G_3_N*_x_*G_3_N*_x_*G_3_N*_x_*G_3_) is not satisfactory, certainly where the size of loop 2 is concerned. However, whether or not irregular RNA G4 motifs possessing loops 1 or 3 that are longer than the classical 1–7 nt are able to fold correctly remains unknown. This report describes the characterization of unusual G-quadruplexes that possess either a long loop 1 or 3 that are found in the 5′- and the 3′-UTRs of human mRNAs.

## Results

### 

#### 

##### Identification of Potential Long Loop 1 and 3 Containing RNA G-quadruplexes

To find irregular PG4 sequences possessing either a long loop 1 or a long loop 3, new python scripts were created and used to search through RefSeq mature transcripts. In the search for a long loop 1, the motif was defined by G*_x_*N_1–40_G*_x_*N_1–7_G*_x_*N_1–7_G*_x_* where G stands for guanine, N can be any nucleotide (A, G, C, U), and *x* ≥ 3 (see Fig. 1). It should be noted that loops 2 and 3 were not limited to 1 nt, but rather were kept at the classical potential size of 1–7 nt. In the search for PG4 sequences with a long loop 3, the sequence motif used was G*_x_*N_1–7_G*_x_*N_1–7_G*_x_*N_1–40_G*_x_* (Fig. 1). This analysis identified 33,651 potential long loop 1 G4 (PLL1-G4) and 33,693 potential long loop 3 G4 (PLL3-G4). These entries contain lots of redundant hits due primarily to the presence of isoforms that can be found in many mRNAs. To provide a more relevant evaluation of the number of PG4 hits, a filter was applied so as to remove the multiple identical hits caused by the isoforms and to thus narrow down the number of sequences selected. The filter reduced the number of hits by almost half, to 17,196 distinct PLL1-G4 and 17,289 distinct PLL3-G4. Supplemental Files S-1 and S-2 present all of the retrieved PG4 with their accession numbers, their sequences, and their positions on the RefSeq mRNA.

In addition, the consecutive guanines over consecutive cytosines (cG/cC) score was calculated for each entry (27), taking into account the 25 nt located both upstream and downstream of the PG4 from the wild-type sequence of an mRNA species. This scoring system has recently been developed to help with the prediction of the formation of a G4 motif (27). Briefly, the guanine residues of a PG4 sequence must be primarily single-stranded to interact with each other and to fold into a G4 motif. Therefore, consecutive residues located in the neighboring regions can potentially impair G4 folding, instead favoring the formation of stable Watson-Crick base pairs with the guanine residues. Following this rational, a cG/cC scoring system was developed (27) in which longer G-tracks should favor G4 folding, whereas longer C-tracks should hinder it. The cG and cC are, respectively, the sum of all of the values attributed to guanine and cytosine residues for a given sequence. Thus, the higher the cG/cC score, the better the probability of the folding into a G4 motif. Considering a threshold of 3.05, which was determined after both a statistical analysis and an in-solution probing of several PG4 candidates (20, 27), 6457 distinct PLL1-G4 (corresponding to 37.5%), and 7157 distinct PLL3-G4 (corresponding to 41.4%) sequences can be proposed to fold into G4 motifs with a certain degree of confidence.

##### In Vitro Folding of PLL1-G4 and PLL3-G4

From the *in silico* search, a subset of PG4 candidates were retained for further studies. Ten PLL1-G4 (Table 1) and 10 PLL3-G4 (Table 2) were manually selected based on the cG/cC scores (the favorable candidates possessing a score of >2.0 and the unfavorable ones a score of <2.0; Ref. 27) from the files in supplemental Files S-1 and S-2. In addition, a mix of candidates with different long loop lengths as well as different locations (3′- *versus* 5′-UTR) was desired. Finally, any candidates possessing guanine-rich regions located either upstream or downstream of the identified G4 that could complicate any interpretation were not retained.

**TABLE 1:** **Characteristics of the manually selected potential long loop 1 G4 used for the in-line probing experiments** Lowercase letters refer to the untranslated regions, whereas the capital letters refer to the coding regions of the mRNA.

Gene	Refseq name	Length of the UTR	Position within the UTR	Length	G-track	L1 (length)	L2	L3
**5′UTR**								
BNIP1	NM_013978	104	7	30	ggg	cgcugccccgagacu (15)	u	ga
DDX43	NM_018665	344	190	29	ggg	auagagagcgu (11)	c	g

**3′UTR**								
AVPR1B	NM_000707	340	61	33	ggg	cacuggaaaugagagcu (17)	a	uaa
B3GNT8	NM_198540	226	45	55	ggg	gccggccccuggcucagccccuccuuccaggucuugau (38)	a	agga
CYSRT1	NM_199001	255	18	65	ggg	ccaggacccagacuucagcaaauguggcucacacagugcc (40)	acaugcc	acaugc
DAG1	NM_004393	2419	1612	54	ggg	aggaaugccuuucgcaauaauguauccauucccugauuga (40)	u	u
DUSP15	NM_001012644	336	189	52	ggg	ccggccugcugcagccaccuggugccuuaguccuu (35)	cu	gga
KIF26A	NM_15656	1101	119	40	ggg	agucucagagaggagacggagugu (24)	gga	a
SPHK2	NM_001243876	633	477	54	ggg	gccggcgcuaggauuugcacuaauguuccucuccccgc (38)	u	ggc
TADA3	NM_006354	393	−6	55	ggg	UAGcccucaccccugccucaggcugauuaucuggccua (38)	ga	gaa

**TABLE 2:** **Characteristics of the manually selected potential long loop 3 G4 used for the in-line probing experiments**

Gene	Refseq name	Length of the UTR	Position within the UTR	Length	G-track	L1	L2	L3 (length)
**5′-UTR**								
GRIA1	NM_000827	365	297	41	ggg	aaa	g	aaacaccaaaucuaugauuggaccu (25)
RNF111	NM_001270530	279	151	61	ggg	agugu	ugag	auuucucucccacuuccgacucucccuagagucucaggau (40)
TEF	NM_003216	116	69	43	ggg	gggc	c	ggaggcgaggugcgcgagccgagucc (26)

**3′-UTR**								
DCTN5	NM_032486	6603	5366	43	ggg	a	a	aggaagcaagcaagugaacaaaugagucu (29)
DOK1	NM_001197260	437	91	33	ggg	u	a	gccaugcugugugagacca (19)
MTF1	NM_005955	5569	3473	54	ggg	u	u	uuuuccugagagacuuuguauaaugcugaauguguccaga (40)
PLXNB1	NM_001130082	632	250	58	ggg	aguu	gacuaa	cuuccagagaguggcuggaagagacuccaggccccu (36)
PTPRU	NM_133178	1152	965	30	ggg	u	u	aaggucucuuuaaaau (16)
STRIP2	NM_020704	2569	95	42	ggg	cucuucu	ggcucuu	ccuaaagauggugcaa (16)
TNRC6C	NM_018996	3990	3775	54	ggg	aacucg	gacagga	cagcacaguguaagcuaaagcccugugua (29)

All of the candidates were tested by in-line probing for their ability to fold in a G4 structure. This technique has been previously used to study G4 folding *in vitro*, and a detailed methodology has already been published (16, 20, 28, 29). Briefly, in-line probing is based on the tendency that RNA has to be differentially hydrolyzed according to its structure (30). The phosphodiester bonds of the RNA molecule are susceptible to slow cleavage through the in-line attack of the 2′-oxygen of the adjacent phosphorus group. This attack occurs when the 2′-oxygen, the phosphorus, and the adjacent 5′-oxygen adopt an in-line conformation that allows the 2′-oxygen to act as a nucleophile and to efficiently cleave the RNA linkage. Thus, the relative rate of spontaneous cleavage is directly related to the surrounding structure of each RNA linkage. The flexible nucleotides (those found in single-stranded regions) are free to adopt any conformation, including the in-line geometry and, consequently, are more susceptible to cleavage. So when the RNA adopts a G4 structure, the nucleotides located in the loops become more flexible (or single-stranded), and an in-line attack by the 2′-oxygen that results in the cleavage of the RNA is possible. On the contrary, the guanines involved in the G4 remain inflexible as they are stabilized by hydrogen bonds of the quartet and less cleavage is detected for these nucleotides.

**FIGURE 2.**
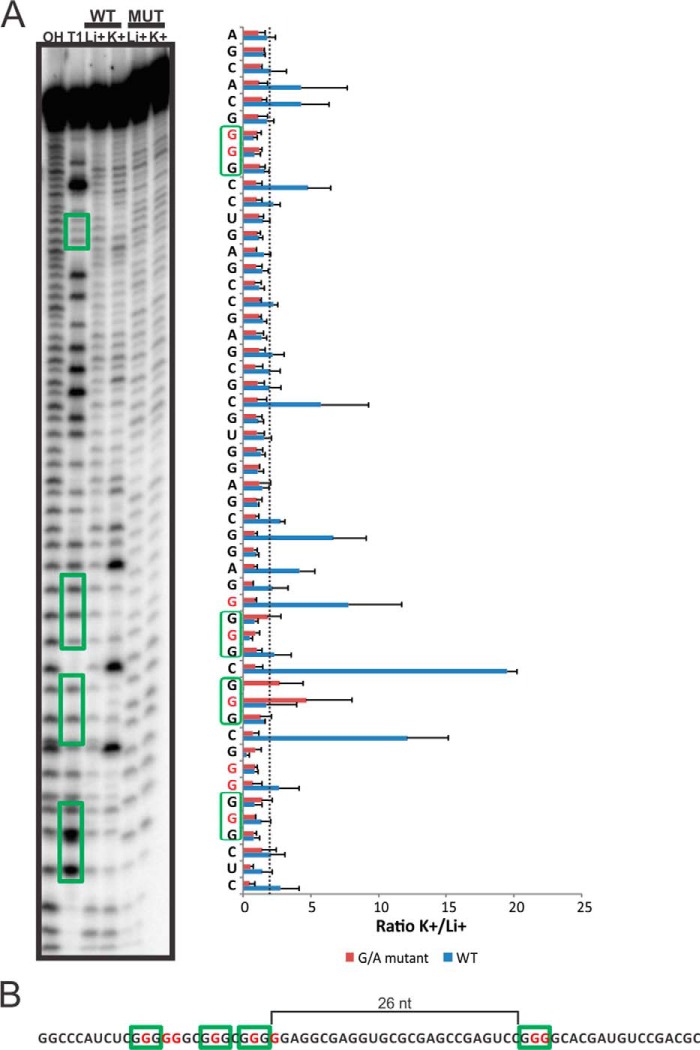
**In-line probing of the TEF candidate.** *A*, autoradiogram of a 10% denaturing (8 m urea) polyacrylamide gel of the in-line probing of the 5′-labeled TEF WT and G/A-mutant PG4 versions performed in the presence of 100 mm of either LiCl or KCl. The lanes designated *OH* and *T1* are an alkaline hydrolysis and an RNase T1 mapping of the WT version, respectively. The guanines thought to being involved in the G4 formation are indicated by the *green boxes*. To the *right of the gel*, the histogram of the K^+^/Li^+^ ratios of the band intensities for each nucleotide of both the TEF wild type and the G/A mutant is shown. The K^+^/Li^+^ ratios are shown in *blue* for the TEF WT and in *red* for the TEF G/A mutant. The *green-boxed guanines* represent the predicted G-tracks. The *dotted line* represents the 2-fold threshold that denotes a significant gain in flexibility. The sequence is indicated on the *y* axis. The *red Gs* on the *y* axis are those mutated to As in the G/A mutant version. Each *bar* represents the average of two independent experiments, and the *error bars* represent the S.D. *B*, the transcript of TEF used for the in-line probing. This candidate possesses a long loop 3 of 26 nucleotides. The G-tracks are denoted by the *green boxes*, and the guanines that are mutated to adenines in the G/A mutant version are in *red*.

The RNA molecules, including a few nucleotides located both upstream and downstream of the PG4 sequences of each candidate, were synthesized to mimic the natural genomic context. In addition to the wild-type (WT) sequences, mutated counterparts in which some key guanines were substituted for adenines (G/A mutants) were also synthesized for each potential candidate. The G/A mutant served as a negative control for G4 formation. All RNA molecules were 5′-end-labeled and, after purification, subjected to in-line probing in the presence of either 100 mm KCl, which favors G4 folding, or 100 mm LiCl, which does not favor G4 formation. An example of an in-line probing gel is shown in Fig. 2*A*, where the PLL3-G4 candidate found in the 5′-UTR of the thyrotrophic embryonic factor (TEF) mRNA was probed. This candidate possesses a loop 1 of 4 nt, a loop 2 of 1 nt, and a long loop 3 of 26 nt (Fig. 2*B*). In the presence of LiCl, no difference was observed between the WT and the G/A mutant, as expected (Fig. 2*A*). Conversely, in the presence of KCl, the increased intensities observed for some of the bands for the WT sequence, as compared with those of the G/A mutant, corresponded to residues located in the loops. Specifically, the single nucleotide of loop 2 was clearly notable, and a few nucleotides of both the loop 1 and the long loop 3 also showed increased intensities. Some residues of the long loop 3 may be involved in secondary and tertiary interactions that render them less susceptible to the in-line attack. For the G/A mutant version of this candidate, the structure remains the same regardless of the ion present (Fig. 2*A*, *lanes* indicated by *MUT*). This result is also clearly visible in the histogram that was obtained by analysis of the banding patterns using the SAFA software (31) in which the ratios of the band intensities (K^+^/Li^+^) are shown for each nucleotide of both the WT and the G/A mutant versions.

**FIGURE 3.**
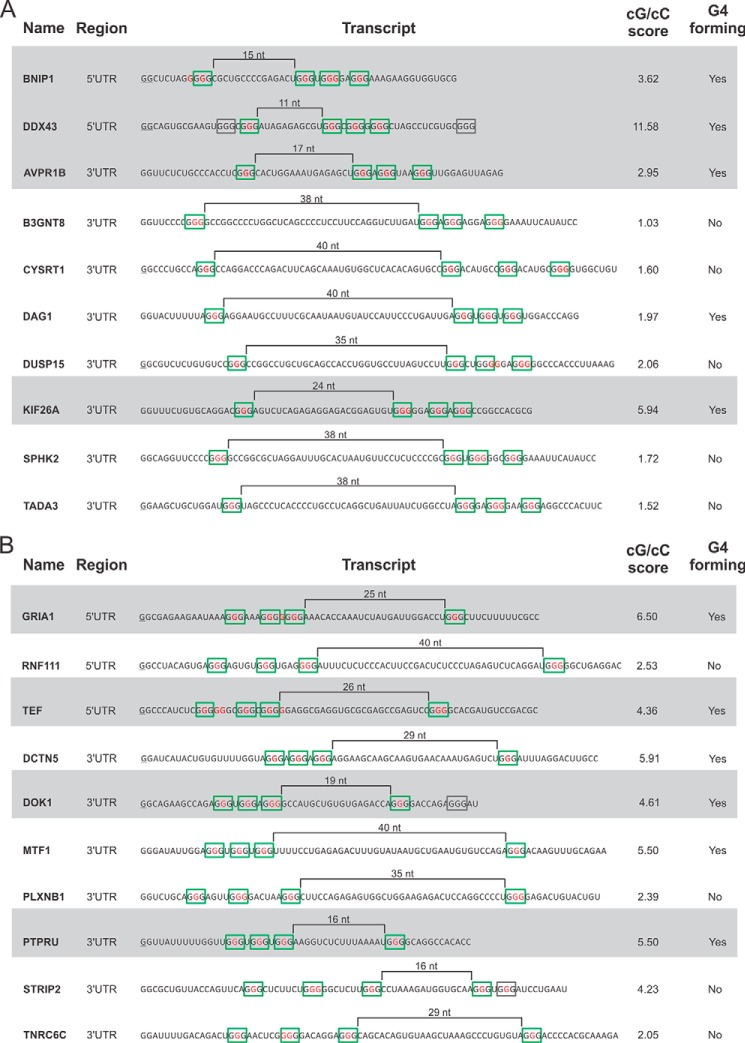
**List of the PLL1-G4 (*A*) and PLL3-G4 (*B*) transcripts tested by in-line probing.** The entire sequence of each transcript is shown along with their respective location on the mRNA. The length of each long loop is indicated over *black brackets*, and the stretches of guanines thought to be involved in the formation of the G4 are indicated by the *green boxes*. Other G-tracks present in the transcripts are identified by the *gray boxes*. The *red guanines* are those that changed to adenosines in the mutant version. The cG/cC scores are also shown (*i.e.* the value calculated for the transcript), and the result of the in-line probing is denoted by *Yes* or *No*. The candidates with a *gray background* are those retained for the *in cellulo* luciferase assays.

A PG4 candidate was considered positive for G4 folding if the K^+^/Li^+^ ratio exceeded an arbitrary threshold of 2 for nucleotides located in the loops of the structure (see the *dotted line* on the *histogram*). This threshold was determined after the in-line probing of several dozen candidates (27). Of the 10 PLL1-G4 candidates tested, 5 adopted a G4 structure in solution according to the in-line probing data, whereas 6 of the 10 PLL3-G4 candidates were positive (Fig. 3). These results are in relatively good agreement with the cG/cC score data. As previously reported, the cG/cC threshold used to predict the folding of a potential G4 was between 2.05 and 3.05. More specifically, the cG/cC scores that fall between these values are ambiguous calls, but for those over and below these limits, the predictions are quite accurate when they are tested *in vitro*. All of the PG4 candidates in this “ambiguous bracket” (like DUSP15, PLXNB1, RNF111, and TNRC6C that have cG/cC scores of 2.06, 2.39, 2.53, and 2.05, respectively) did not adopt a G4 motif according to in-line probing. The only exceptions were DAG1, which had a cG/cC score of 1.97 and adopted a G4 structure, and STRIP2, which had a score of 4.23 and surprisingly was not able to fold in a G4. The denaturing gels as well as the histogram of each candidate tested are shown in supplemental File S-3.

##### In Cellulo Translational Modulation by PLL1-G4 and PLL3-G4

From the subset of 11 candidates folding into a G4 motif (five PLL1-G4 and six PLL3-G4 candidates) according to in-line probing, 8 were selected for further testing to determine whether or not they can modulate translation *in vivo*. Of these eight PG4 candidates, four were located in the 5′-UTR, two included a long loop 1, and two included a long loop 3 (see Fig. 3, specifically the candidates shaded with a *gray background*). Of the four candidates where the G4 is located in the 3′-UTR and whose formation was detected in solution, two were selected because they included a long loop 1, whereas two others were selected because they included a long loop 3 (see Fig. 3, specifically the candidates with a *gray background*).

**FIGURE 4.**
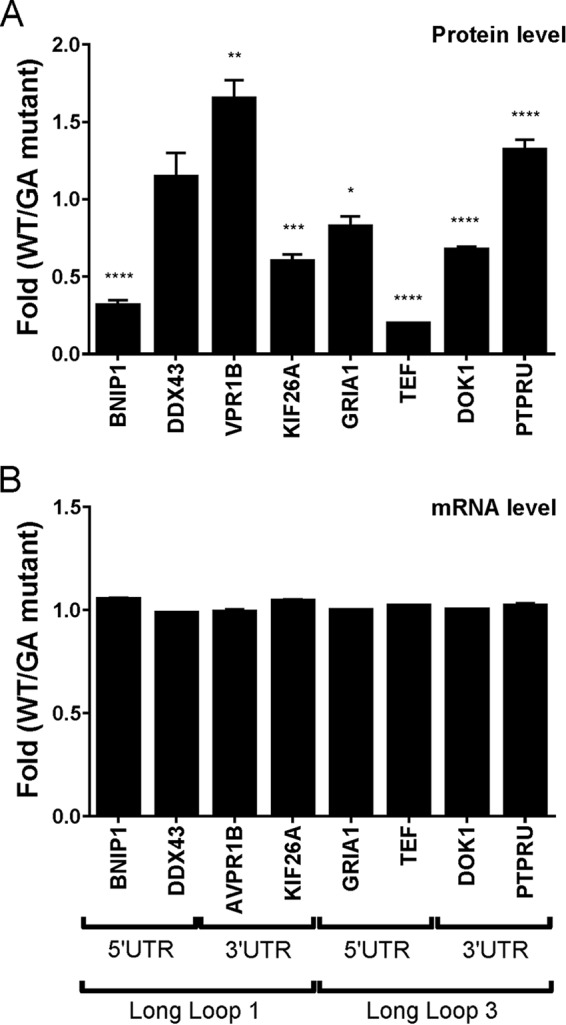
**Gene expression levels of the constructs containing the different PLL1-G4 and PLL3-G4 candidates (located in either the 5′ or the 3′-UTRs) fused to the luciferase gene at the protein (*A*) and the mRNA levels (*B*).** The *x* axis identifies the different candidates used, and the *y* axis identifies the fold difference (*i.e.* WT result divided by G/A-mutated result). *Error bars*, mean ± S.D. The *p* values are *p* = 0.0116 (*), *p* = 0.0026 (**), *p* = 0.0002 (***), and *p* < 0.0001 (****). The experiments were repeated three times (on three different days), and each time, three technical replicates were performed.

For each candidate, the full-length sequence of the UTR was cloned either upstream (for the 5′-UTR) or downstream (for the 3′-UTR) of the Renilla luciferase gene. In each case, a G/A mutant version was also constructed. The guanines mutated are those identified in *red* in Fig. 3. The resulting clones were separately transfected into HEK293 cells. The transfected cells were grown for 24 h, the cells were recovered and lysed, and the luciferase assays were performed. The Renilla luciferase:Firefly luciferase ratio was determined for each lysate, and that of the WT sequence was divided by that of its G/A mutant counterpart (Fig. 4*A*). The gene expression levels of almost all of the candidates were either significantly positively or negatively affected. Looking at the candidates located in the 5′-UTR, regardless of the type of long loop, the G4 of both TEF and BNIP1 exhibited the greatest translational modulation (0.19- and 0.31-fold, respectively), whereas the effect of the GRIA1 G4 was only modest at 0.82. DDX43 was the only G4 without a significant alteration in the gene expression levels. Interestingly, this candidate is also the only one from those tested *in cellulo* that did not stall the Superscript III enzyme when the full 5′-UTR was used as a template in a primer extension reaction (data not shown).

With the exception of the latter, all of the G4 motifs located in the 5′-UTR decreased the expression level of the luciferase gene (BNIP1, GRIA1, and TEF). To ensure this effect was independent of the transcription level, the amount of mRNA present for every candidate tested was determined by quantitative PCR (qPCR; Fig. 4*B*). No variation was observed, thus confirming that the decreases observed resulted from the repression of translation and not from a transcriptional effect.

For the G4 retrieved within the 5′-UTR of GRIA1 and DDX43, each include a potential initiation codon AUG suggestive of an upstream open reading frame or an extended reporting coding sequence. Translation from the initiation codon within the 5′-UTR of DDX43, for instance, would produce a peptide of a mere three amino acids. This seems highly improbable, and if it were to occur the effect would be expected to be similar for the WT as well as the G/A mutant. More importantly, when we look at the raw data from the luciferase assays, the number of counts detected for DDX43 and GRIA1 are in the same range as the other candidates without an AUG codon in their 5′-UTR (that is to say BNIP1 and TEF; raw data not shown). This indicates that any potential initiation codons within the 5′-UTR of GRIA1 and DDX43 would not significantly alter the luciferase reporter gene expression. The situation was more variable for the G4 motifs located in the 3′-UTR (Fig. 4*A*). The folding of the G4 found in the AVPR1B and PTPRU mRNAs significantly increased the levels of luciferase expression (1.65- and 1.31-fold, respectively). Conversely, the G4 motif of both the KIF26A and the DOK1 mRNAs decreased the luciferase levels 0.59- and 0.67-fold, respectively. More importantly, all of the candidates tested exhibited a significant effect on the expression levels of the Renilla luciferase in the presence of the G4 motifs. Once again, there was no variation at the mRNA level, indicating that the observed differences were not due to a variation at the transcription level but strictly to one at the translation level.

## Discussion

The growing evidence of the importance of RNA G4 in gene regulation makes these structures very attractive as potential therapeutic targets. In fact it has been shown that it is possible to control the formation of the G4 located in the 5′-UTR of a histone variant associated with cellular differentiation (H2AFY) by using small antisense oligonucleotides that can bind directly onto the G4 itself or onto the surrounding regions (17). The consequence of such oligonucleotide binding is that it enables the disruption of the G4 structure, acting as a *bona fide* gene-regulation control switch at the translational level. The discovery of G4s with a long loop (>7 nt) suggests that the very structure adopted by the long loop could become a convenient platform for the binding of protein factors that may be usefully targeted by an array of chemicals to enhance the specific recognition of a G4; hence, gaining a better understanding of their stability, structure, and composition is imperative. The recent report of the presence of natural irregular RNA G4 with long central loops of up to 70 nt in length and 2 short loops that are limited to 1 nt each (20) in the 5′-UTRs of certain mRNA prompted the design of scripts searching for other natural irregular G4 motifs in human mRNAs. A script was designed to find irregular G4s with either a long loop 1 or a long loop 3 of up to 40 nt in size located in both the 5′ and 3′-UTRs. This report shows that G4-forming sequences are considerably more varied than expected and are not limited to the canonical sequence G_3_N_1–7_G_3_N_1–7_G_3_N_1–7_G_3_ even though synthetic irregular RNA G4s such as G_3_N_15_G_3_N_15_G_3_N_15_G_3_ have been shown to be stable enough to form *in vitro* (26).

Biophysical approaches such as circular dichroism are not helpful in the characterization of such a G4 sequence because the structural heterogeneity of the long RNA (specifically the long loop) interferes with the classical G4 spectrum. Consequently, in-line probing is a more suitable technique with which to characterize G4. An added bonus of the use of the in-line probing technique is that only trace amounts of the RNA samples are required. A subset of PG4 candidates was in-line-probed in solution, and the resulting data (Fig. 3) confirmed that the cG/cC score is a reasonable tool for the prediction of G4 folding (27). With the exception of both DAG1 and STRIP2, all of the other 18 PG4 candidates had cG/cC scores and in-line probing data in good agreement. It is interesting to note that BNIP1, AVPR1B, and KIF26A, which are PLL1-G4 candidates, and GRIA1 and TEF, which are PLL3-G4 candidates, possessed, in addition to a very large loop, a short loop that is longer than one nucleotide. This shows that the initial belief that the folding of a G4 motif that includes a long loop requires the other loops to be restricted to 1 nt is not an absolute prerequisite (20). That said, all of the other candidates possessing two short loops longer than one nucleotide that were tested were not able to adopt a G4 conformation even though they were all had a low cG/cC score.

The PG4 candidates were tested *in cellulo* for their abilities to modify the translation levels when their respective UTRs were fused to the luciferase mRNA. A total of 8 candidates were tested along with their respective G/A mutants (Fig. 4). For seven of these candidates, the folding of the G4 motif significantly affected the translation level of the luciferase mRNA. As shown in Fig. 4*B*, the observed effect was clearly not due to a variation at the transcriptional level. With the exception of the DDX43 candidate, which showed no effect, all of the other G4 motifs located in the 5′-UTR repressed translation. Many examples of canonical G4-forming sequences have been described, and the most common effect reported at the translation level was an inhibitory action (28, 32) through interference with the recruitment of a preinitiation complex that delayed translation. The G4 itself may also bind inhibitors, which in turn hinder the translational process (25). A few examples of up-regulation at the translation level have been reported for G4s found in the 5′-UTR (33–35); although none has been observed in this study, the possibility that irregular G4 may exert this kind of effect cannot be excluded.

The variations observed for the PG4 motifs located in the 3′-UTR were less drastic than those observed for the candidates located in the 5′-UTR. Two candidates repressed translation (KIF26A and DOK1), whereas two others enhanced it (AVPR1B and PTPRU). The regulation of gene expression at the translational level by G4 located in the 3′-UTR can be explained primarily by 3′-end processing (16, 36, 37). For example, the G4 located in the 3′-UTR can increase the efficiencies of the polyadenylation of alternative sites, leading to the expression of either shorter or longer transcripts (25). When the high-throughput sequencing data of RNA from five tissues (brain, liver, kidney, testis, and muscle), which are mapped on the UCSC genome browser, were used to detect potential alternative polyadenylation sites, none was found in the 3′-UTRs of the four candidates studied here. The Expression and Polyadenylation Database (xPAD) (38), a comprehensive map of >1 million polyadenylation sites in major cancers and tumor cell lines, was also searched for any alternative polyadenylation sites located in the areas surrounding the G4 studied here, but none was found. Alternatively, the modulation of translation resulting from the presence of a G4 motif located within a 3′-UTR may also interfere with the miRNA regulatory network of a specific mRNA (16). For example, a search through the experimentally validated microRNA-target interactions database (miRTarBase) (39) found a link between the DOK1 mRNA and miR-218. In fact, this miRNA has 3 binding sites in the DOK1 3′-UTR, 2 of which are located 6 nt both upstream and downstream of the G4. Although this validation is categorized as “less strong evidence” according to miRTarBase, miR-218 may play a role depending on the status of the G4 (folded or not). Finally, the G4 found in the 3′-UTR may also influence ribosome re-initiation, although this possibility has yet to be reported. Clearly, the up- and down-regulation of translation by 3′-UTR G4 motifs appears to be a more complex situation than that of those located in the 5′-UTR, and the mechanisms involved remain unknown. One way to properly address this issue might be to develop a relatively large collection of both regular and irregular 3′ UTR G4 motifs exerting effects on translation and to systematically investigate any potential mechanism(s); that, however, falls outside of the scope of this study.

In conclusion, irregular G4 forming sequences with either a long loop 1 or a long loop 3 can modulate gene expression at the translational level. The analysis presented here focused on motifs located in the 5′ and 3′-UTRs, but one cannot exclude the possible impact of the presence of these irregular G-rich sequences in the open reading frame. As was recently shown by Endoh and Sugimoto (40), a G4 can act as a roadblock to translation when it is located in a specific reading frame. Importantly, the definition of the sequences that form the G4 motif needs to be revisited. The prediction of an RNA molecule structure should be more accurate when the global sequence including the regions bordering a PG4 sequence is considered. With this in mind, the G4Hunter tool appears to be an excellent starting point for future studies (41).

## Experimental Procedures

### 

#### 

##### In Silico Analysis

The bioinformatic search for potential G4 (PG4) with long loops 1 or 3 required a new script, as the available ones were inappropriate. A python script was used to search the RefSeq mature transcripts obtained from the UCSC table browser on August 18, 2014 for the regular expression of 4 identical series of at least 3 guanines possessing either loop 1 or loop 3 of 1–40 nt in size and the other two loops of 1–7 nt in size (G*_x_*N_1–40_G*_x_*N_1–7_G*_x_*N_1–7_G*_x_* and G*_x_*N_1–7_G*_x_*N_1–7_G*_x_*N_1–40_G*_x_*, respectively, where G represents guanine, N can be any nucleotide, and *x* ≥ 3). Only non-overlapping PG4 were kept to narrow down the number of sequences; however, multiple duplicates were found due to the presence of redundant transcripts such as the different isoforms of mRNAs. The script also provided the cG/cC score of the PG4 calculated using the 25 nt located both upstream and downstream of the sequences of interest (27). The resulting spreadsheets are available insupplemental Files S-1 and S-2 for the candidates possessing long loops 1 and 3, respectively.

##### RNA Synthesis and 5′-Labeling of Both the PG4 and the Corresponding Mutants for In-line Probing

All RNA molecules used for in-line probing were synthesized by *in vitro* transcription using purified T7 RNA polymerase as previously described (29). Briefly, two oligodeoxynucleotides (ODNs) (2 mm each, Life Technologies) were annealed together, and then purified *Pfu* DNA polymerase was used in PCR reactions in the presence of 5% DMSO to fill in the gaps. One ODN was complementary to the PG4 sequence, with the addition of 17-nt complementary to the T7 RNA polymerase promoter at the 3′-end, whereas the other ODN corresponded to the sequence of the T7 RNA polymerase promoter. See S-4 in supplemental File S456 for a complete list of all ODNs used. The resulting duplex DNA products were then ethanol-precipitated, washed with 70% ethanol, and dissolved in ultrapure water. Run-off transcriptions were then performed in a final volume of 100 μl using purified T7 RNA polymerase in the presence pyrophosphatase (0.01 U; Roche Diagnostics) and 5 mm concentrations of each nucleotide triphosphate in a buffer containing 80 mm HEPES-KOH (pH 7.5), 24 mm MgCl_2_, 40 mm dithiothreitol, and 2 mm spermidine. The reactions were incubated for 2 h at 37 °C followed by DNase RQ1 (Promega, Madison, WI) treatment for 30 min at 37 °C. The RNA was then purified by phenol:chloroform extraction and recovered by ethanol precipitation. The RNA products were then fractionated by denaturing (8 m urea) 8% polyacrylamide gel electrophoresis. The RNAs in the gels were detected by ultraviolet shadowing, and the bands corresponding to the appropriate sizes were excised from the gels. The transcripts were then eluted overnight at room temperature in a buffer containing 1 mm EDTA, 0.1% sodium dodecyl sulfate, and 0.5 m ammonium acetate, ethanol-precipitated, dried, and dissolved in water, and their concentrations were then determined by spectrometry at 260 nm using a NanoDrop spectrophotometer (Thermo Fisher Scientific, Mississauga, ON). The 5′-ends of the gel-purified RNAs were then dephosphorylated using 50 pmol of RNA in reactions containing 1 unit of Antarctic phosphatase with the supplied buffer (New England BioLabs, Pickering, ON) in a final volume of 10 μl. After 30 min of incubation at 37 °C, the enzyme was heat-inactivated for 10 min at 70 °C. The dephosphorylated RNAs (10 pmol) were then 5′-radiolabeled with 6 units of T4 polynucleotide kinase (Affymetrix-USB, Cleveland, OH) for 1 h at 37 °C in the presence of 2 μl of [γ-^32^P]ATP (6000 Ci/mmol; PerkinElmer Life Sciences) in a final volume of 10 μl. The reactions were stopped by the addition of 2 volumes of formamide loading dye (95% formamide, 10 mm EDTA, and 0.025% xylene cyanol), and the resulting samples were run on 8% polyacrylamide (8 m urea) denaturing gels. The bands were detected by autoradiography, excised from the gels, and recovered as described above.

##### In-line Probing

The detailed methodology and step-by-step protocol of the in-line probing of G4 candidates has been described previously (29). Briefly, 50,000 cpm (<1 nm) of 5′-end labeled RNA of both the WT and the G/A mutant were incubated in a final volume of 10 μl for 5 min at 70 °C and then were slowly cooled to room temperature over 1 h in a buffer containing 20 mm Li-cacodylate (pH 7.5) and 100 mm concentrations of either LiCl or KCl. After the folding step, the volume of each sample was adjusted to 100 μl such that the final concentrations were always 20 mm Li-cacodylate (pH 8.5), 20 mm MgCl_2_, and 100 mm concentrations of either LiCl or KCl. The reactions were then incubated for a period of 40 h at room temperature followed by an ethanol precipitation of the RNA in the presence of glycogen. The precipitated RNA was washed with 70% ethanol and then was dissolved in 30 μl of denaturing formamide loading buffer. Two ladders were prepared for the in-line probing experiment, specifically an alkaline hydrolysis (mapping of each nucleotide of the sequence) and an RNase T1 digestion of the transcripts (mapping of the guanines). For the alkaline hydrolysis ladder, 50,000 cpm of 5′-end-labeled WT RNA (<1 nm) were dissolved in 5 μl of water, 1 μl of 2N NaOH was added, and the reaction was incubated at room temperature for 1 min before quenching by the addition of 3 μl of 1 m Tris-HCl (pH 7.5). The RNA molecules were then ethanol-precipitated and dissolved in 20 μl of formamide loading buffer. For the RNase T1 ladder, 50,000 cpm of 5′-end-labeled WT RNA (<1 nm) were dissolved in 9 μl of buffer containing 20 mm Tris-HCl (pH 7.5), 10 mm MgCl_2_, and 100 mm LiCl. The mixtures were incubated at 37 °C for 2 min in the presence of 0.6 unit of RNase T1 (Roche Diagnostics), and the reactions were then quenched by the addition of 20 μl of formamide loading buffer. All samples and ladders were quantified using a single well gamma particle counter (Bioscan QC-2000) and then were equilibrated to have equivalent amounts of radioactivity loaded into each well. The equilibrated samples and ladders were then fractionated on denaturing (8 m urea) 10% polyacrylamide gels. After electrophoresis, the gels were dried, and the bands were visualized by exposure to a phosphor screen (GE Healthcare) using a Typhoon Trio imaging system (GE Healthcare). The SAFA software was used to quantify the individual band intensities (31). The intensities of the bands formed in the presence of KCl (conditions favorable for G4 formation) was then divided by the intensities of the corresponding bands formed in the presence of LiCl (conditions unfavorable for G4 formation). The results are presented as one representative gel, and a bar graph of the means and S.D. of K^+^/Li^+^ band intensity ratios were obtained from two independent experiments.

##### DNA Cloning and Plasmid Constructions

All restriction enzymes used were purchased from New England BioLabs. The complete 5′-UTR sequences of the WT and the G/A mutants of the BNIP1, DDX43, GRIA1, and TEF candidates and the complete 3′-UTR sequences of the WT and the G/A-mutants of the AVPR1B, KIF26A, DOK1, and PTPRU candidates were cloned into the psiCHECK 2 vector (Promega). Prior to cloning into psiCHECK 2, three restriction sites (SpeI, SalI, and SacI) were inserted downstream of the NheI site so as to create more cloning possibilities for the 5′-UTR candidates, generating the vector psiCHECK 2.1 (6312 bp). The Q5 site-directed mutagenesis kit (New England BioLabs) was used according to the manufacturer's instructions with the forward ODN being 5′-gtcgaccggtccggagctcACCATGGCTTCCAAGGTG-3′ and the reverse ODN being 5′-ttaattaaactagtaccggtGGCTAGCCTATAGTGAGTC-3′ (the capital letters represent sequences complimentary to the vector, and the lowercase letters represent the added restriction sites). All constructs were confirmed by DNA sequencing. UTR DNA templates for the PTPRU and KIF26A candidates were synthesized using GeneArt gene synthesis (Thermo Fisher Scientific), whereas AVPR1B, DDX43, DOK1, and GRIA1 were GeneArt strings (Thermo Fisher Scientific). Each candidate and its corresponding G/A mutant sequence (the G/A mutations were the same as those used for the in-line probing constructs) were PCR-amplified using specific ODNs (see S-4 in supplemental File S456 for a complete list of the ODNs used). Shorter UTR sequences such as the TEF and BNIP candidates were generated through PCR reactions using purified *Pfu* DNA polymerase and two overlapping ODNs (2 mm each, Life Technologies). All of the PCR products were separated on 1% agarose gels, and the desired DNAs were then extracted from the gels using the QIAquick Gel Extraction kit (Qiagen, Mississauga, ON) followed by ethanol precipitation and washing with 70% ethanol. The dried pellets of the 5′-UTR DNA molecules were dissolved in water and then digested with restriction enzymes SpeI and SacI for the cloning of DDX43, BNIP1, GRIA1, and TEF upstream of the Renilla luciferase (Rluc) reporter gene of the psiCHECK 2.1 vector. For the 3′-UTR candidates AVPR1B and KIF26A, the PCR products were digested with PmeI and NotI, whereas for DOK1 and PTPRU, XhoI and NotI were used for the insertion downstream of the Rluc reporter gene of psiCHECK 2.1. All sequences were verified by DNA sequencing. For the full-length sequences of all UTRs used see S-6 in supplemental File S456.

##### In Cellulo Luciferase Assays

HEK 293 cells were cultured in Dulbecco's modified Eagle's medium (DMEM, Wisent, St-Bruno, QC) supplemented with 10% fetal bovine serum (FBS, Wisent) and 1 mm sodium pyruvate (Wisent) at 37 °C in a 5% CO_2_ and 100% H_2_O atmosphere. Twenty-four hours pretransfection, 4.5 × 10^4^ cells were seeded in a 24-well plate. The next day, to 50 ng of psiCHECK 2.1 vector (containing the candidates to be tested), 450 ng of pUC19 vector was added as a carrier and transfected into cells using 0.5 μl of Lipofectamine 2000 (Life Technologies) per well. Twenty-four hours later the cells were lysed in passive lysis buffer (Promega), and the luciferase assays were performed following the Dual-luciferase Reporter Assay manufacturer's protocol (Promega) using the Glomax 20/20 luminometer. The Renilla luciferase:Firefly luciferase ratio was determined for each lysate. The ratio obtained for the WT sequence was divided by that of the G/A mutant sequence. The means and S.D. were calculated from at least three independent experiments. Statistical significance was valuated with an unpaired Student's *t* test using the GraphPad Prism 6.07 software.

##### Quantitative PCR

RNA was extracted from whole cells and dissolved in Tripure Isolation Reagent (Roche Diagnostics) according to manufacturer's protocol. After phenol/chloroform extraction and isopropyl alcohol precipitation, the total RNA was dissolved in water and treated with 2 units of DNase RQ1 for 1 h at 37 °C. The DNase was then removed by phenol/chloroform extraction, and the RNA ethanol was precipitated. The resulting RNA pellets were washed with 70% ethanol and dried, and the pellets were dissolved in water. RNA integrity was assessed using an Agilent 2100 Bioanalyzer (Agilent Technologies, Cedar Creek, TX). Reverse transcription reactions were performed on 1.1 μg of total RNA with Transcriptor reverse transcriptase (Roche Diagnostics), random hexamers, dNTPs (Roche Diagnostics), and 10 units of RNAseOUT (Life Technologies) following the manufacturer's protocol (Roche Diagnostics) in a total volume of 10 μl. Specific primers for Fluc (forward, 5′-GTGGGCAAGGTGGTGCCATT-3′; reverse, 5′-AATCATAGGGCCGCGCACAC-3′) and Rluc (forward, 5′-AAGGGCCTCCACTTCAGCCA-3′; reverse, 5′-TTCTTCAGCACGCGCTCCAC-3′) were individually dissolved to 20–100 μm stock solutions in Tris-EDTA buffer (Integrated DNA Technologies, Coralville, IA) and then diluted as primer pairs to 1 μm in RNase and DNase-free water. qPCR reactions were performed in a final volume of 10 μl in 96-well plates on a CFX-96 thermocycler (Bio-Rad) with 5 μl of 2X iTaq Universal SYBR Green Supermix (Bio-Rad), 10 ng (3 μl) of cDNA, and a 200 nm final concentration (2 μl) of the primer pair. The cycling conditions used were: 3 min at 95 °C; 50 cycles of 15 s at 95 °C, 30 s at 60 °C, and 30 s at 72 °C. The CT (threshold cycle) ratios of Rluc/Fluc were calculated for each candidate, and a final ratio of WT/mutant was determined. In every qPCR run, a no-template control was performed for each primer pair, and these were consistently negative. Quantitative PCR assays were performed at the RNomics Platform of the Université de Sherbrooke, Sherbrooke, Québec, Canada.

## Author Contributions

F. B. conducted most of the experiments, analyzed the results, and wrote most of the paper. J.-M. G. conducted the *in silico* analyses. F. A. conducted the experiments on the long loop 1 G-quadruplexes. J.-P. P. conceived the idea for the project, analyzed the results, and wrote the paper with F. B.

## Supplementary Material

Supplemental Data

## Footnotes

4 The abbreviations used are:

G4: G-quadruplex
PLL1-G4: potential long loop 1 G-quadruplex
PLL3-G4: potential long loop 3 G-quadruplex
ODN: oligodeoxynucleotide
cG/cC: consecutive guanines/consecutive cytosines
nt: nucleotide(s)
Rluc: Renilla luciferase
qPCR: quantitative PCR.
