# Supplementary material for: Irregular G-quadruplexes Found in the Untranslated Regions of Human mRNAs Influence Translation
Source: J Biol Chem. 2016 Aug 24;291(41):21751–60. doi: 10.1074/jbc.M116.744839 (PMC5076843; doi:10.1074/jbc.M116.744839)
Supplement: Supplemental Data [file supp_291_41_21751__index.html]

Irregular G-quadruplexes found in the untranslated regions of human mRNAs influence translation — Irregular G-quadruplexes Found in the Untranslated Regions of Human mRNAs Influence Translation — Irregular G-quadruplexes and the Modulation of Translation — Supplemental Data 

# Irregular G-quadruplexes Found in the Untranslated Regions of Human mRNAs Influence Translation

## Supplemental Data

- Supplemental S1 (.xls, 16.2 MB) - Raw data from bioinformatic search for long loop 1 candidates
- Supplemental S2 (.xls, 16.1 MB) - Raw data from bioinformatic search for long loop 3 candidates
- Supplemental S3 (.pdf, 21.4 MB) - Results of in-line probing for all candidates
- Supplemental S4S6 (.docx, 33 KB) - Oligonucleotides and UTR
