# Supplementary material for: Irregular G-quadruplexes Found in the Untranslated Regions of Human mRNAs Influence Translation
Source: J Biol Chem. 2016 Aug 24;291(41):21751–60. doi: 10.1074/jbc.M116.744839 (PMC5076843; doi:10.1074/jbc.M116.744839)
Supplement: Supplemental Data [file 10.1074_M116.744839_jbc.M116.744839-3.pdf]

Supplementary information

Supplemental information S-3

In-line probing of all candidates tested. Autoradiogram of a 10% denaturing (8M urea) polyacrylamide gel of the in-line probing of the 5'-labeled candidate wild-type (wt) and G/A-mutant PG4 versions performed in the presence of 100 mM of either LiCl or KCl. The lanes designated OH and T1 are an alkaline hydrolysis and an RNase T1 mapping of the wt version, respectively. The guanines thought to being involved in the G4 formation are indicated by the green boxes. To the right of the gel, the histogram of the K+ /Li+ ratios of the band intensities for each nucleotide of both the wild type and the G/A-mutant is shown. The K+ /Li+ ratios are shown in blue for the wt and in red for the G/A mutant. The green boxed guanines represent the predicted G-tracks. The dotted line represents the two fold threshold that denotes a significant gain in flexibility. The sequence is indicated on the y-axis. The red Gs on the y-axis are those mutated to As in the G/A mutant version. Each bar represents the average of two independent experiments, and the error bars represent the standard deviations. The transcripts of the candidates used for the in-line probing are shown below. The G-tracks are denoted by the green boxes, and the guanines that are mutated to adenines in the G/A mutant version are in red.

AVPR1B

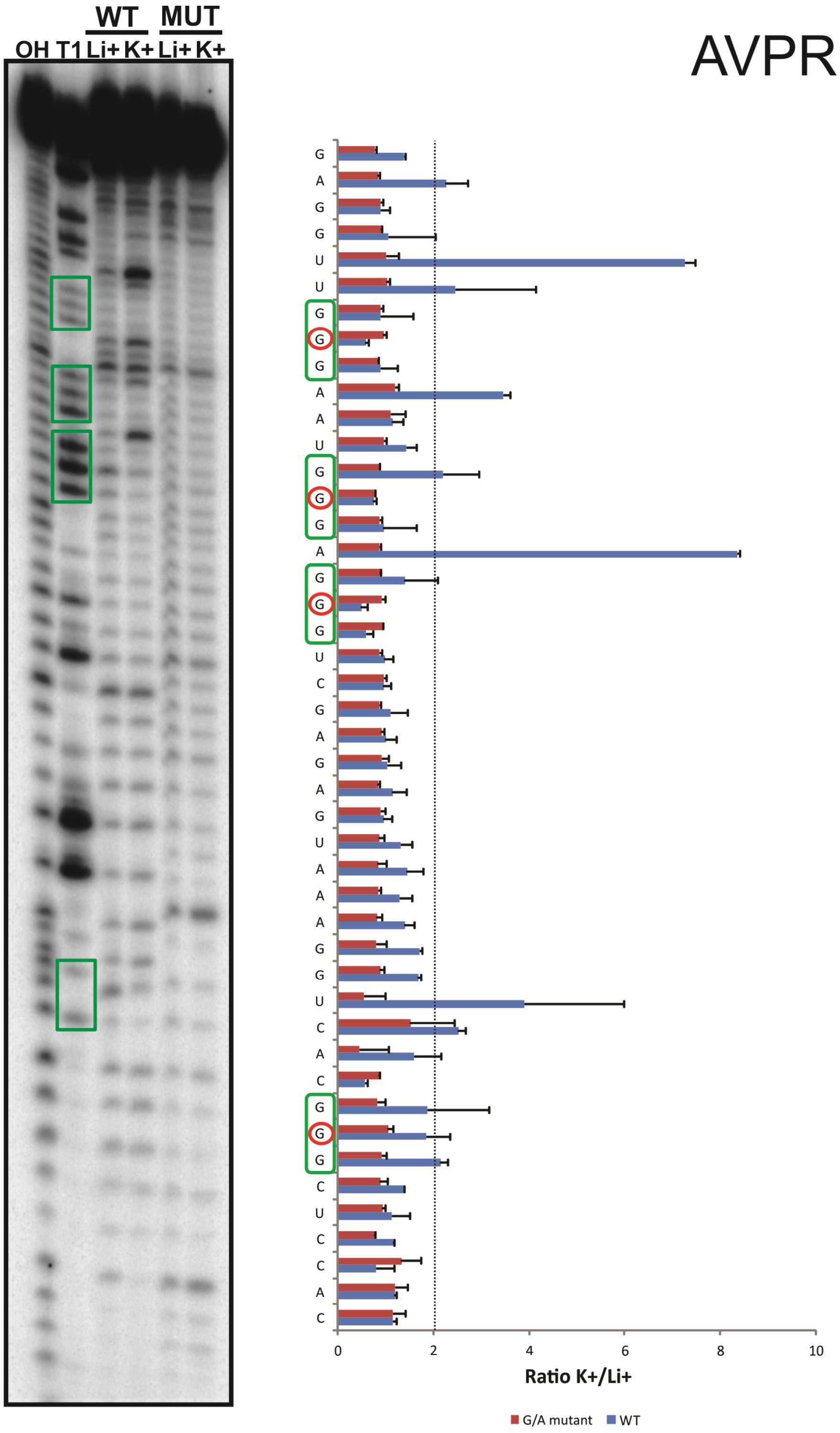

17 nt

GGUUCUCUGCCCACCUCGGGCACUGGAAAUGAGAGCUGGGAGGGUAAGGGUUGGAGUUAGAG 2.95

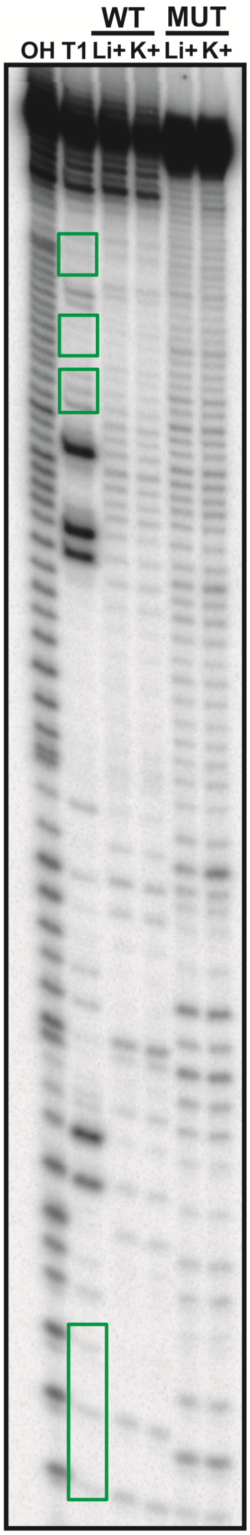

B3GNT8

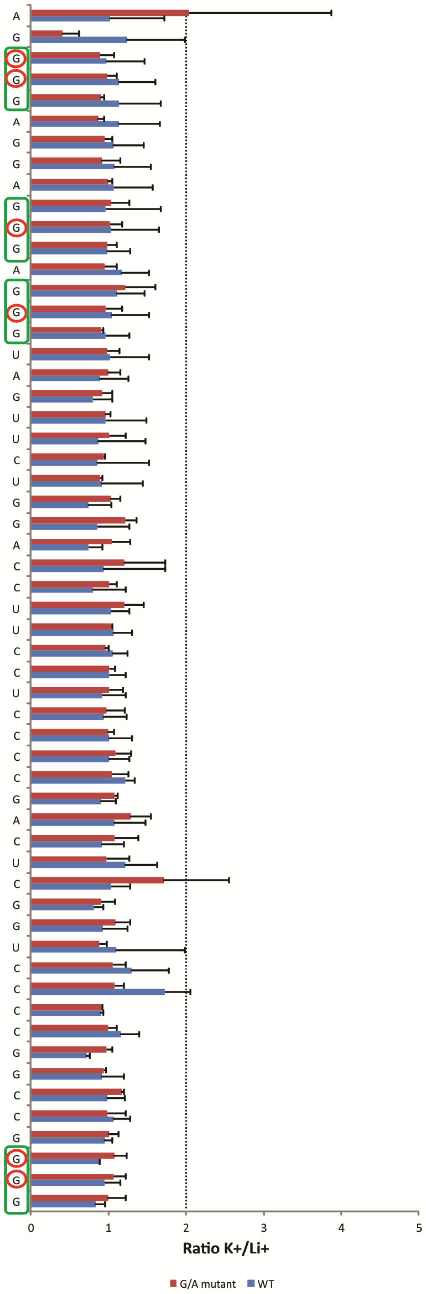

38 nt

GGUUCCCCGGGGCCGGCCCCUGGCUCAGCCCCUCCUUCCAGGUCUUGAUGGGAGGGAGGAGGGGAAAUUCAUAUCC 1.03

BNIP1

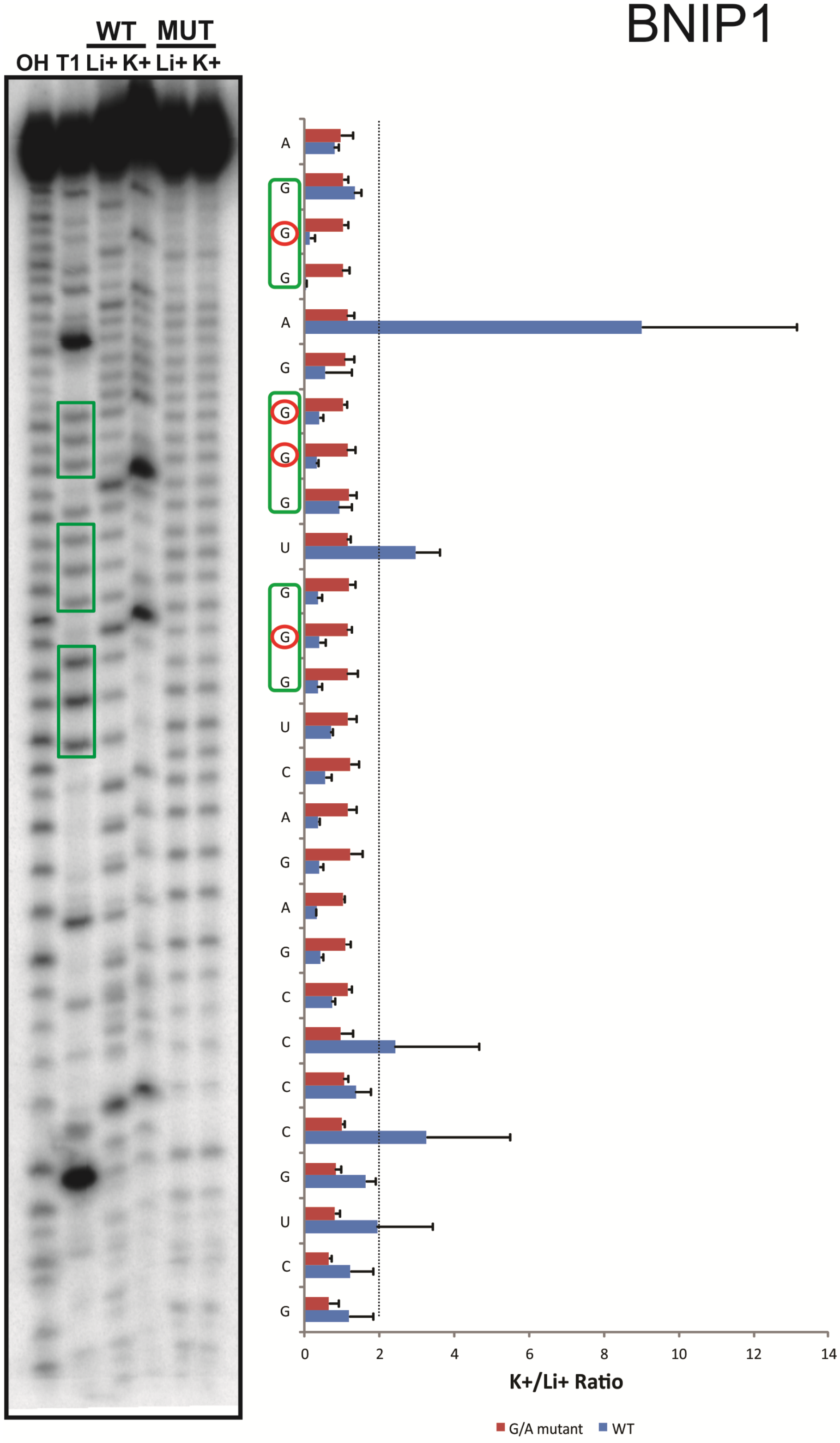

15 nt

GGCUCUAGGGGGCGCUGCCCCGAGACUGGGUGGGGAGGGAAAGAAGGUGGUGCG 3.62

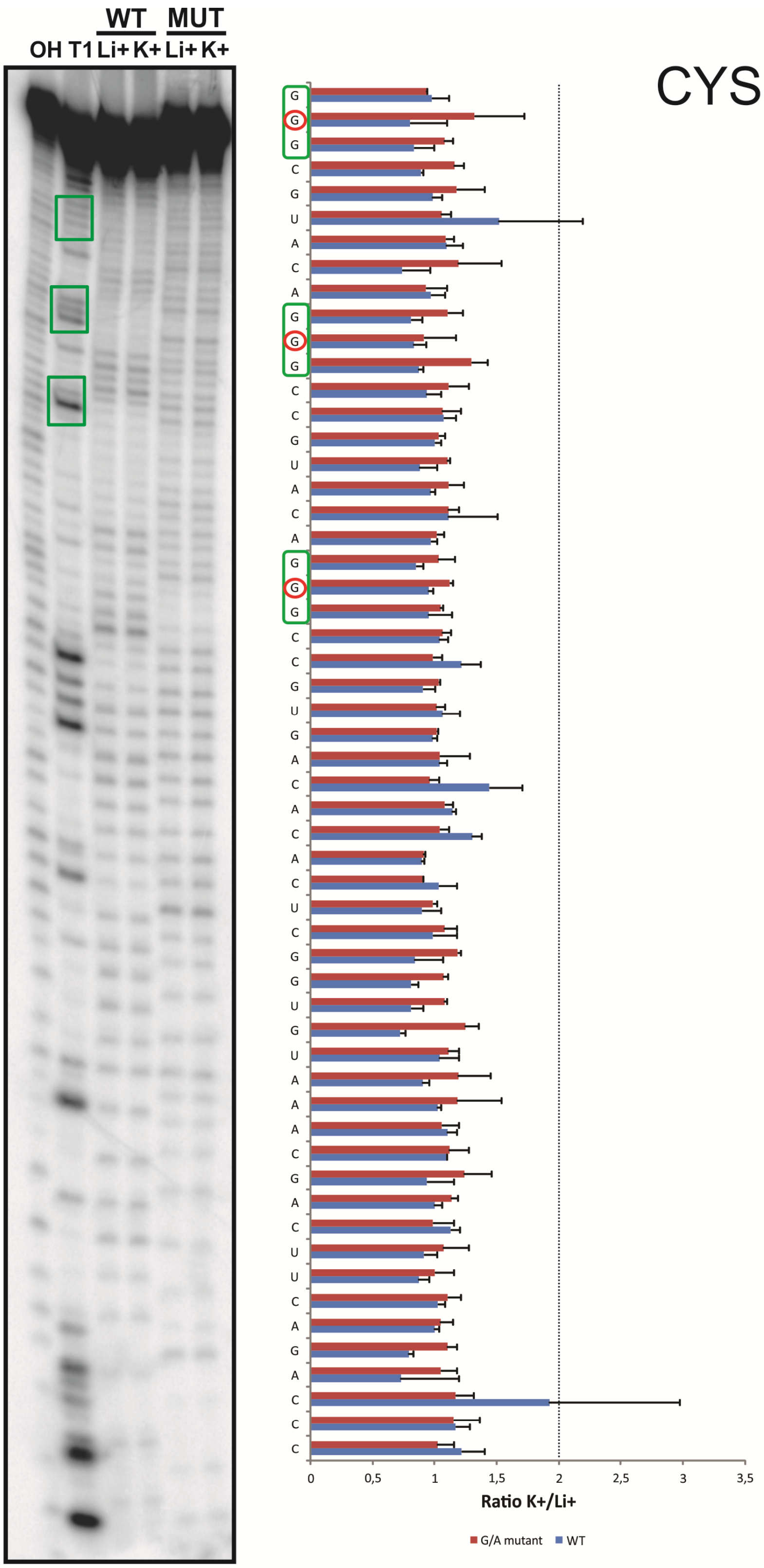

CYSRT1

40 nt

GGCCCUGCCAGGGCCAGGACCCAGACUUCAGCAAAUGUGGCUCACACAGUGCCGGGACAUGCCGGGACAUGCGGGGUGGCUGU 1.60

DAG1

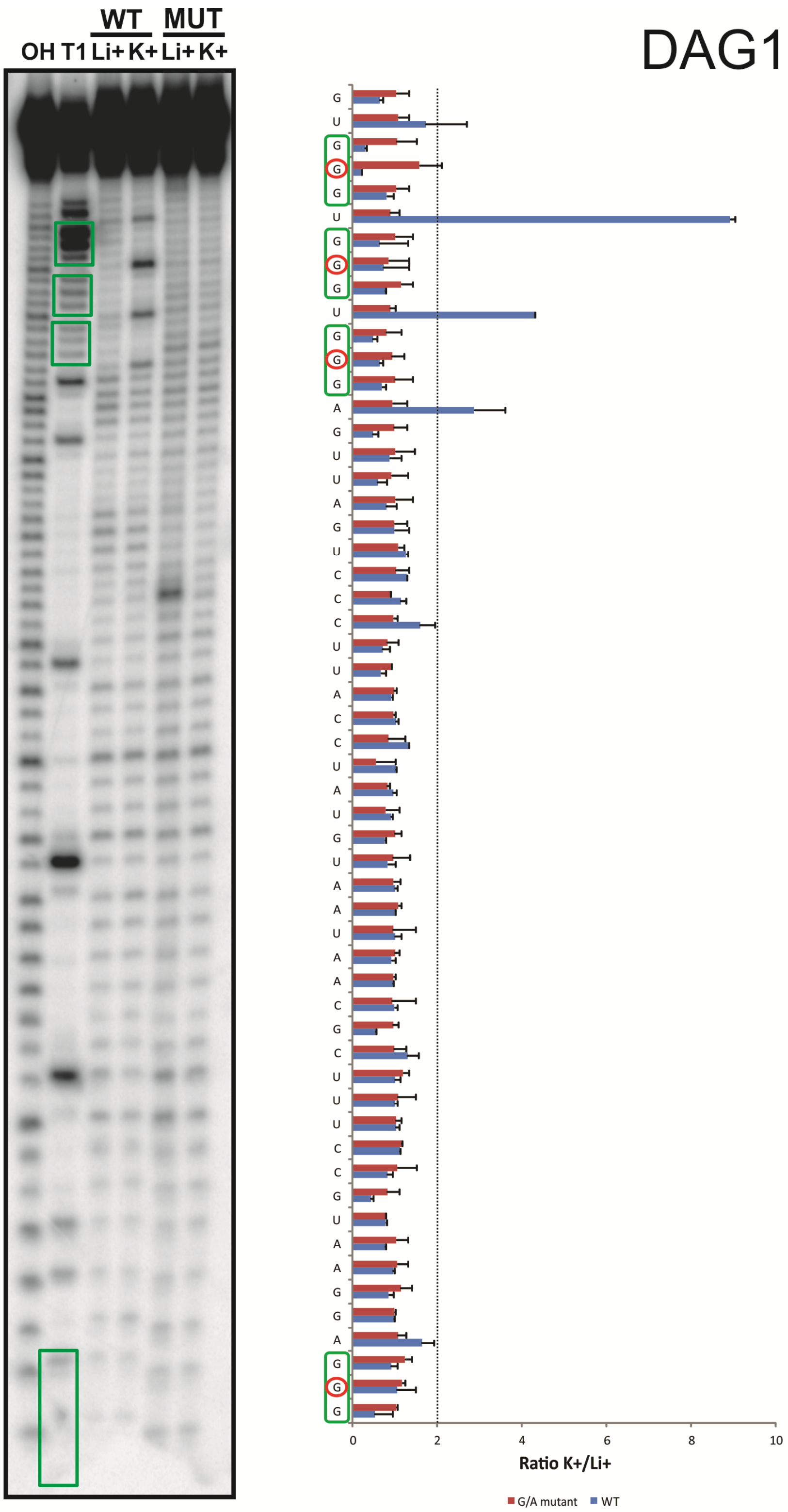

40 nt

GGUACUUUUUAGAGAGGAAUGCCUUUCGCAAUAAUGUAUCCAUUCCCUGAUUGAGAGUGAGUGAGUGGACCCAGG 1.97

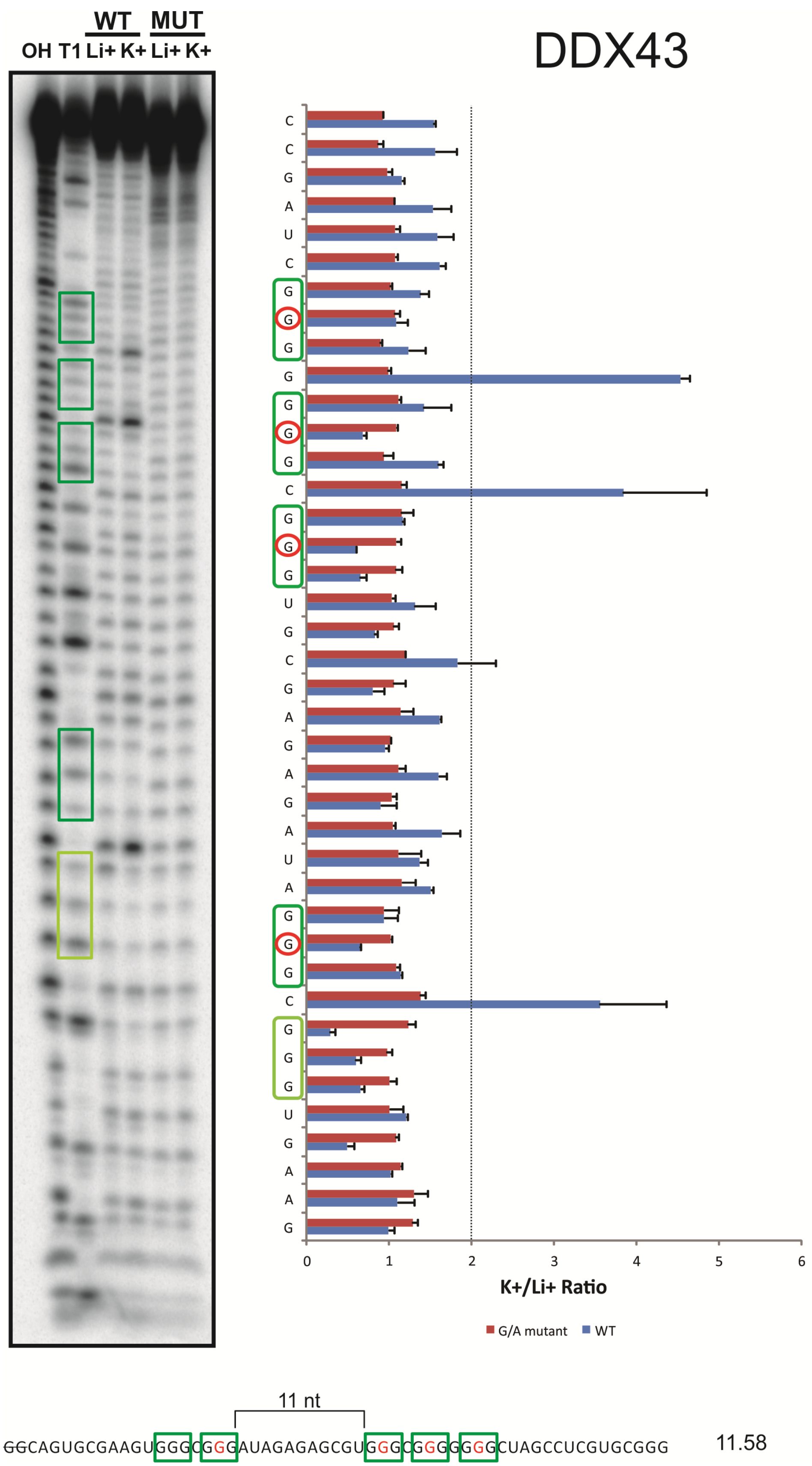

DDX43
WT
MUT
OH T1 Li+ K+ Li+ K+
C C G A U C G G G G G G G C G G G U G C G A G A G A U A G G G C G G G U G A A G
0 1 2 3 4 5 6
K+/Li+ Ratio
G/A mutant
WT
11 nt
GGCAGUGCGAAGUGGGCGGGAUAGAGAGCGUGGGCGGGGGGCUAGCCUCGUGCGGG
11.58

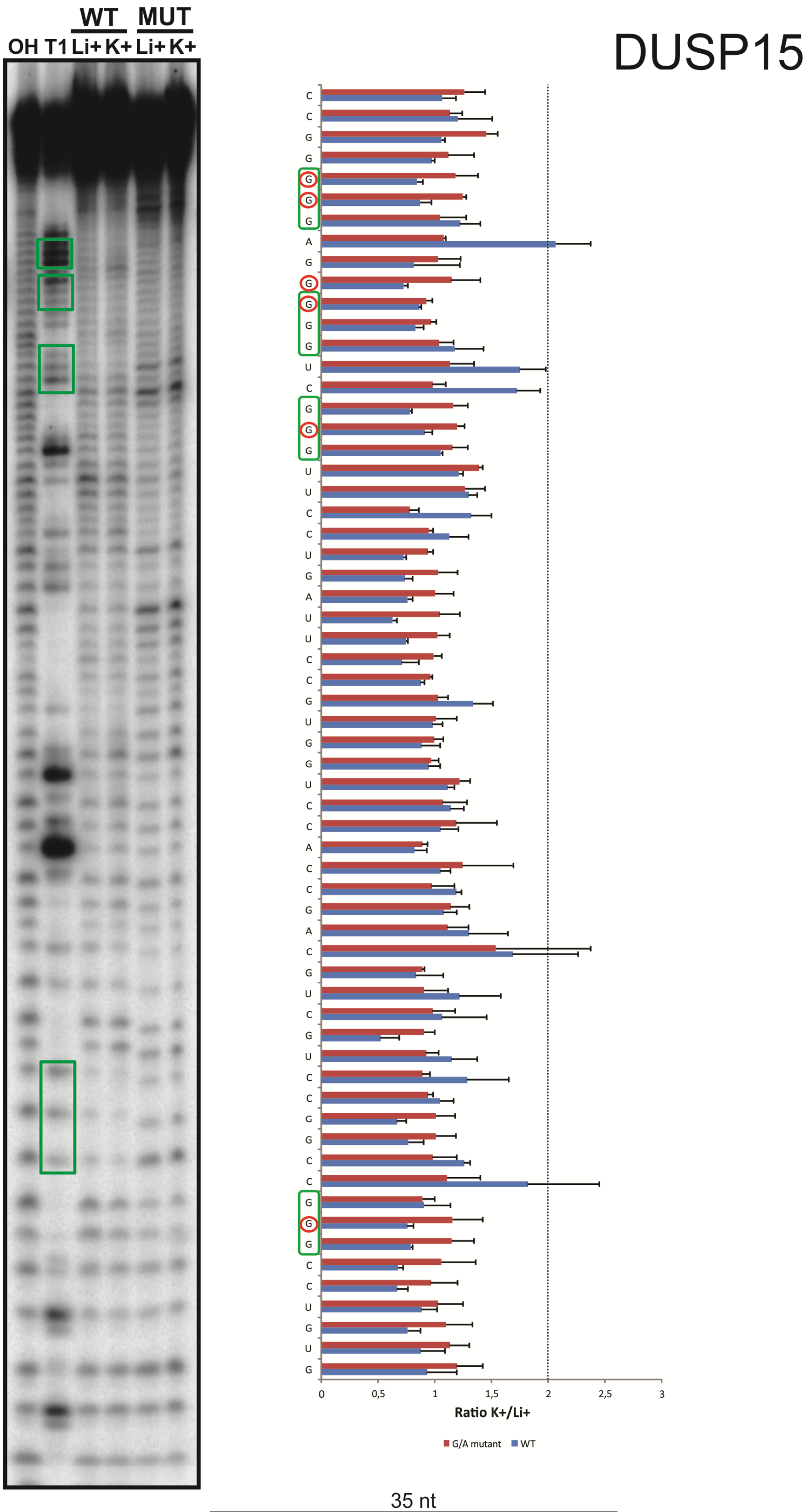

35 nt

GGCGUCUCUGUGUCCGGGCCGGCCUGCUGCAGCCACCUGGUGCCUUAGUCCUUGGGCUGGGGAGGGGGCCCACCCUUAAAG 2.06

KIF26A

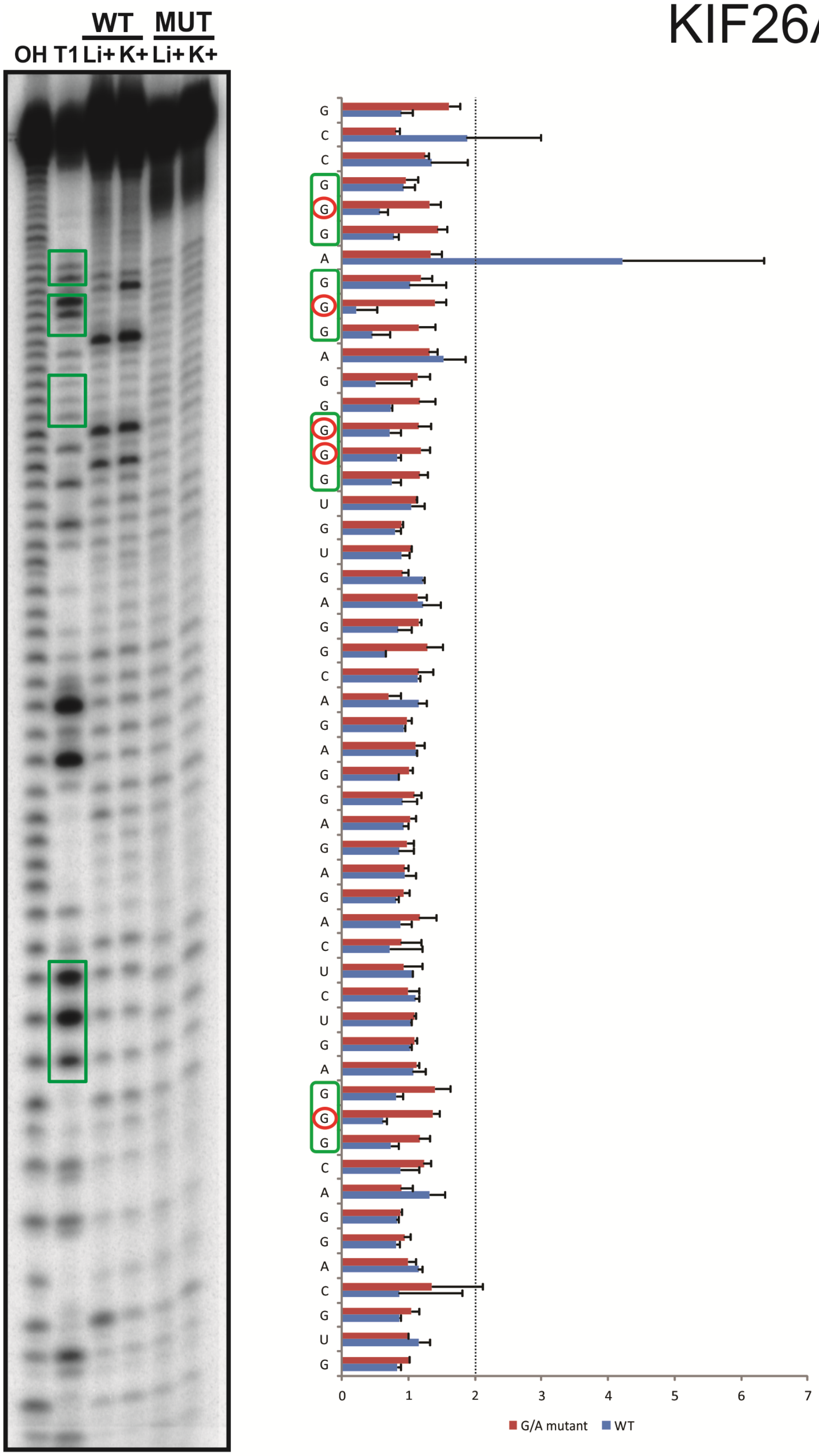

24 nt

GGUUUCUGUGCAGGACGGGAGUCUCAGAGAGGAGACGGAGUGUGGGGGAGGGAGGGCCGGCCACGCG 5.94

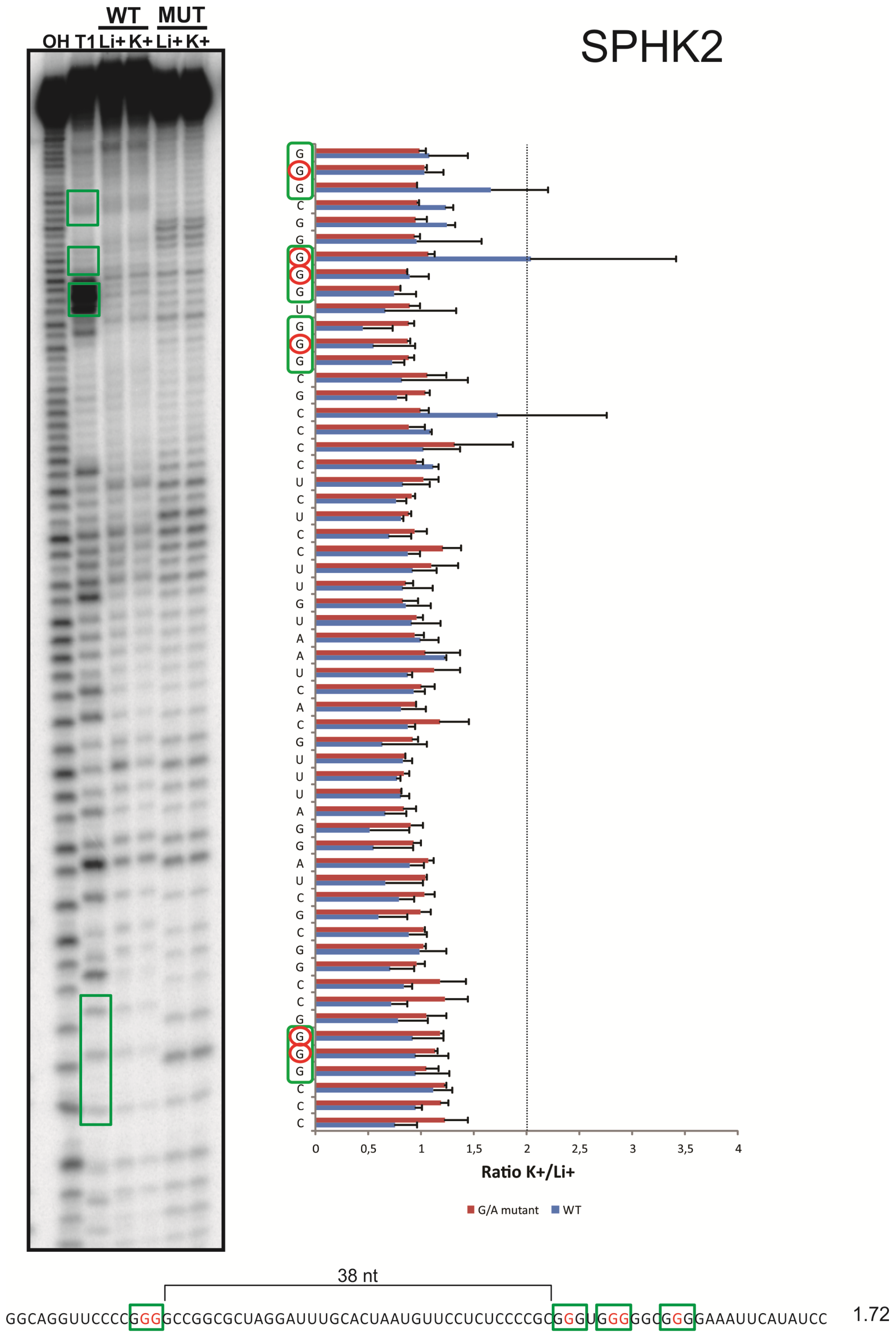
WT MUT
OH T1 Li+ K+ Li+ K+
SPHK2
0 0,5 1 1,5 2 2,5 3 3,5 4
Ratio K+/Li+
G/A mutant WT
38 nt
GGCAGGUUCCCCGGGGCCGGCGCUAGGAUUUGCACUAAUGUUCCUCUCCCCGCGGGUGGGGGCGGGGAAAUUCAUAUCC
1.72

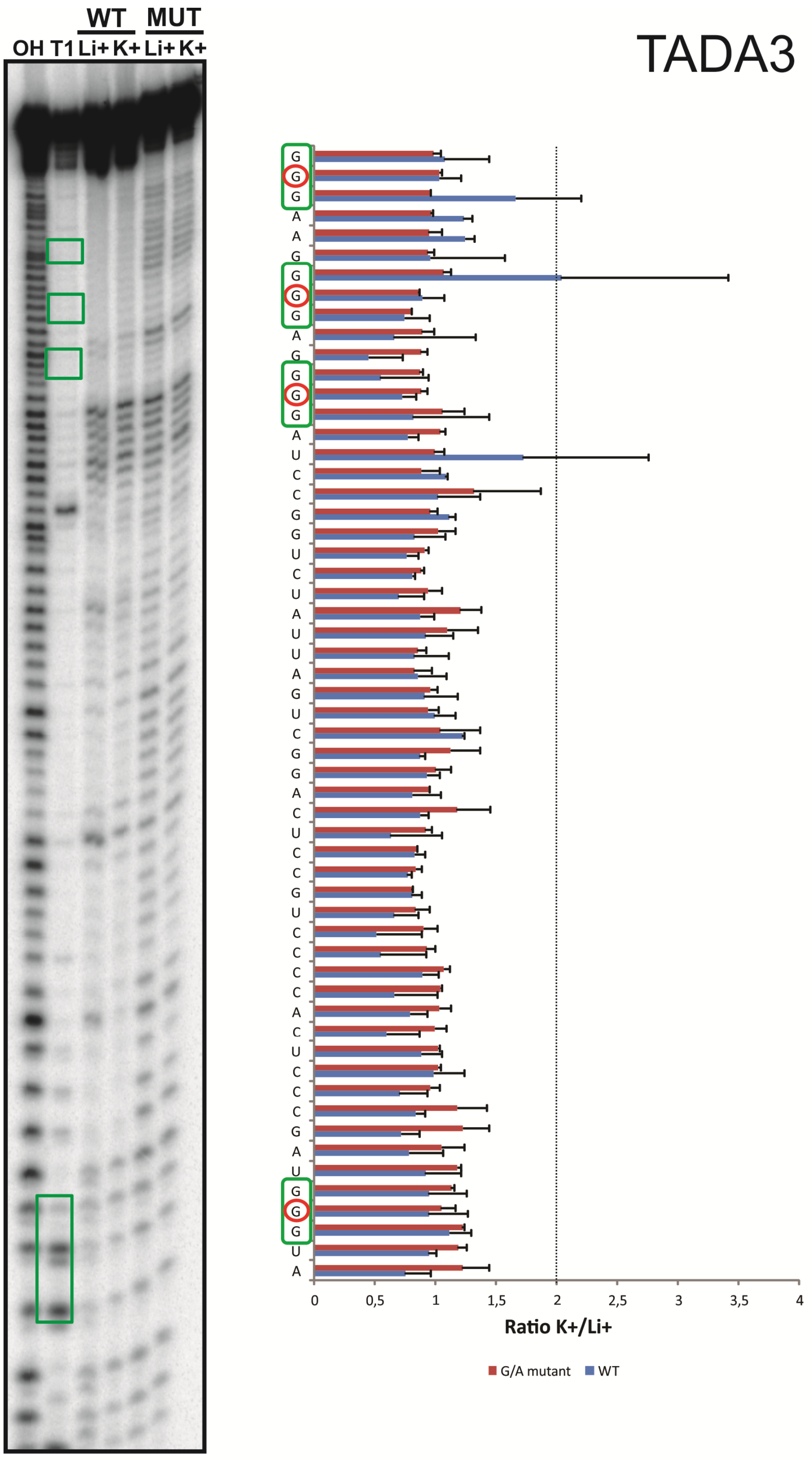

38 nt

GGAAGCUGCUGGAUGGGUAGCCCUCACCCCUGCCUCAGGCUGAUUAUCUGGCCUAGGGGAGGGGAAGGGAGGCCCACUUC 1.52

DCTN5

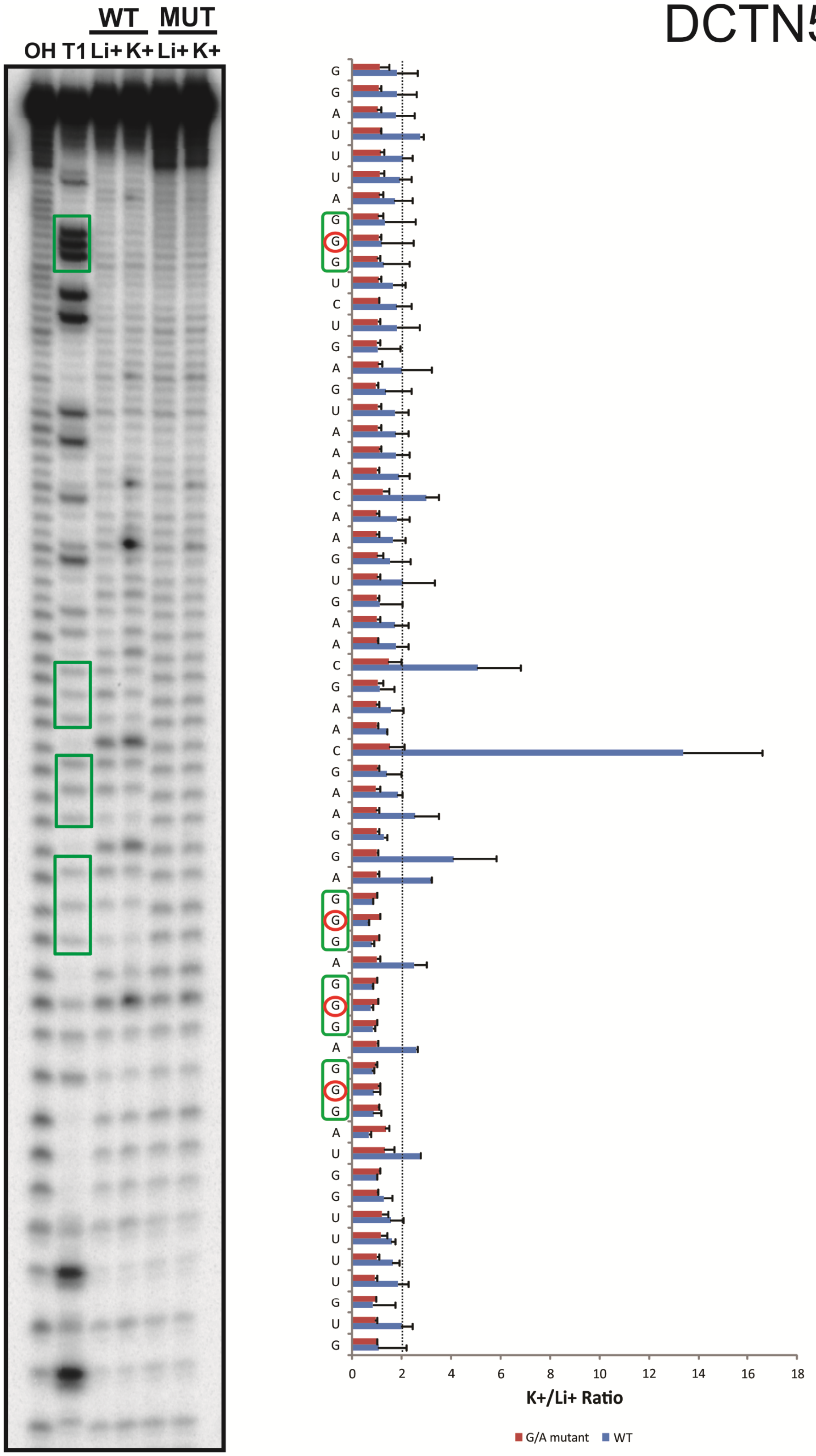

29 nt

~~G~~GAUCAUACUGUGUUUUGGUAGGGAGGGAGGGAGGAAGCAAGCAAGUGAACAAAUGAGUCUGGGAUUUAGGACUUGCC 5.91

DOK1

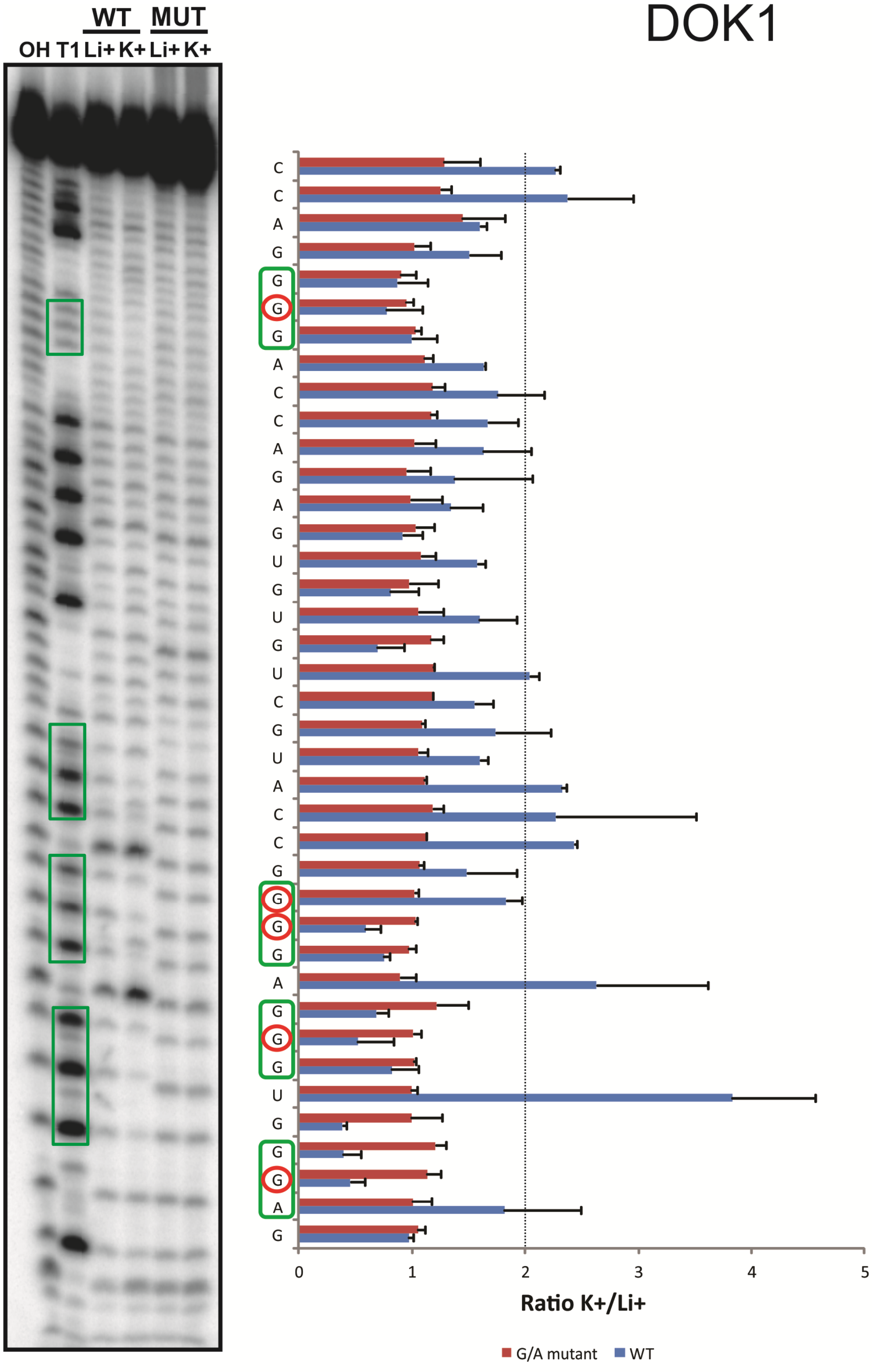

19 nt

GGCAGAAGCCAGAGGGUGGGAGGGGCCAUGCUGUGUGAGACCAGGGGACCAGAGGGAU 4.61

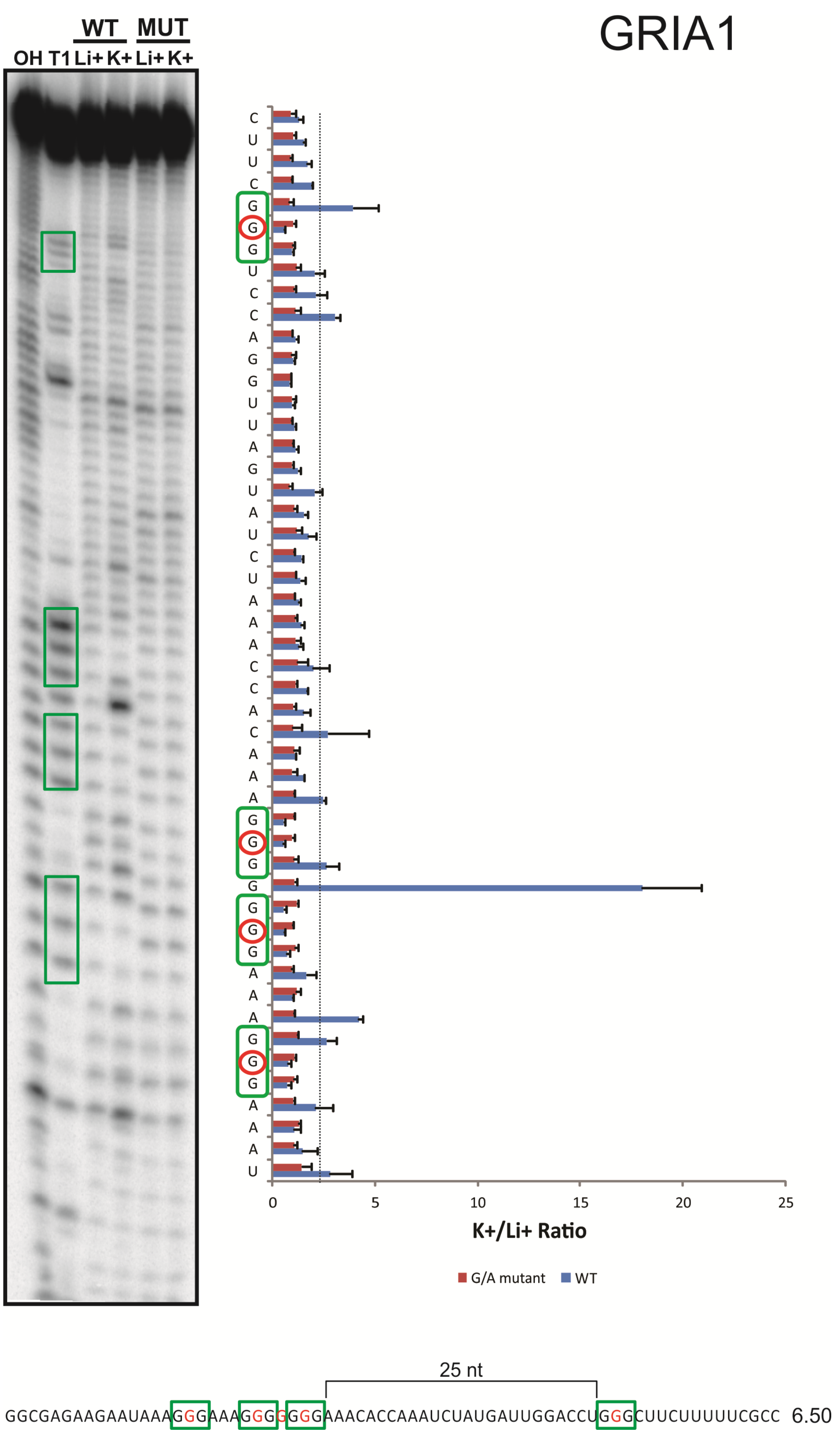
GRIA1
WT MUT
OH T1 Li+ K+ Li+ K+
C U U C G G G U C C A G G U U A G U A U C U A A A C C A C A A A G G G G G G G A A A G G G A A A U
0 5 10 15 20 25
K+/Li+ Ratio
G/A mutant WT
25 nt
GGCGAGAAGAAUAAAGGGAAAGGGGGGGAAACACCAAAUCUAUGAUUGGACCUGGGCUUCUUUUUCGCC 6.50

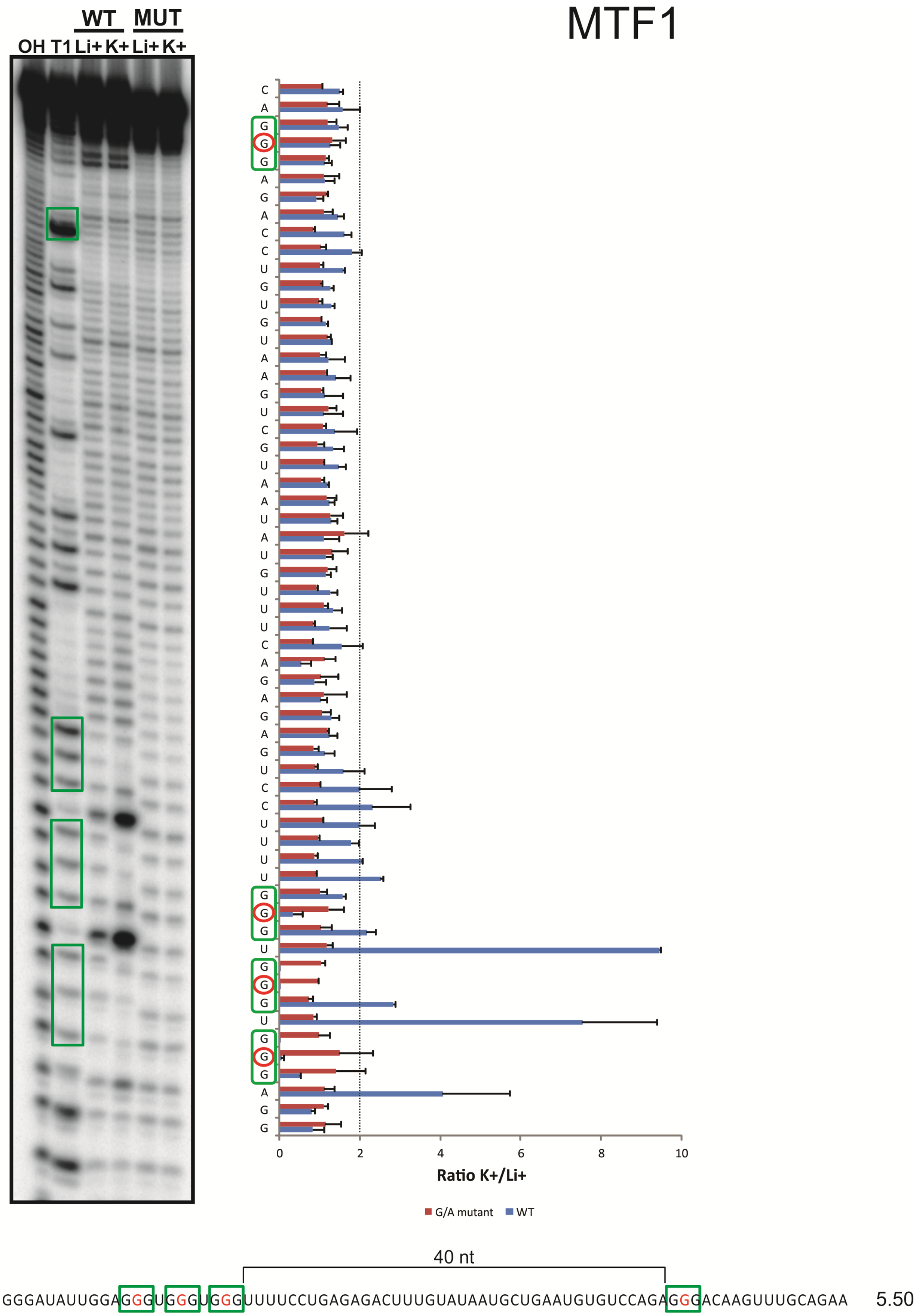

MTF1
WT MUT
OH T1 Li+ K+ Li+ K+
C A G G G A G A C C U G U G U A A G U C G U A A U A U G U U U C A G A G A G U C C U U U U G G G U G G G U G G G A G G
0 2 4 6 8 10
Ratio K+/Li+
G/A mutant WT
40 nt
GGGAUAUUGGAGGGUGGGUGGGUUUUCCUGAGAGACUUUGUAUAAUGCUGAAUGUGUCCAGAGGGACAAGUUUGCAGAA
5.50

PLXNB1

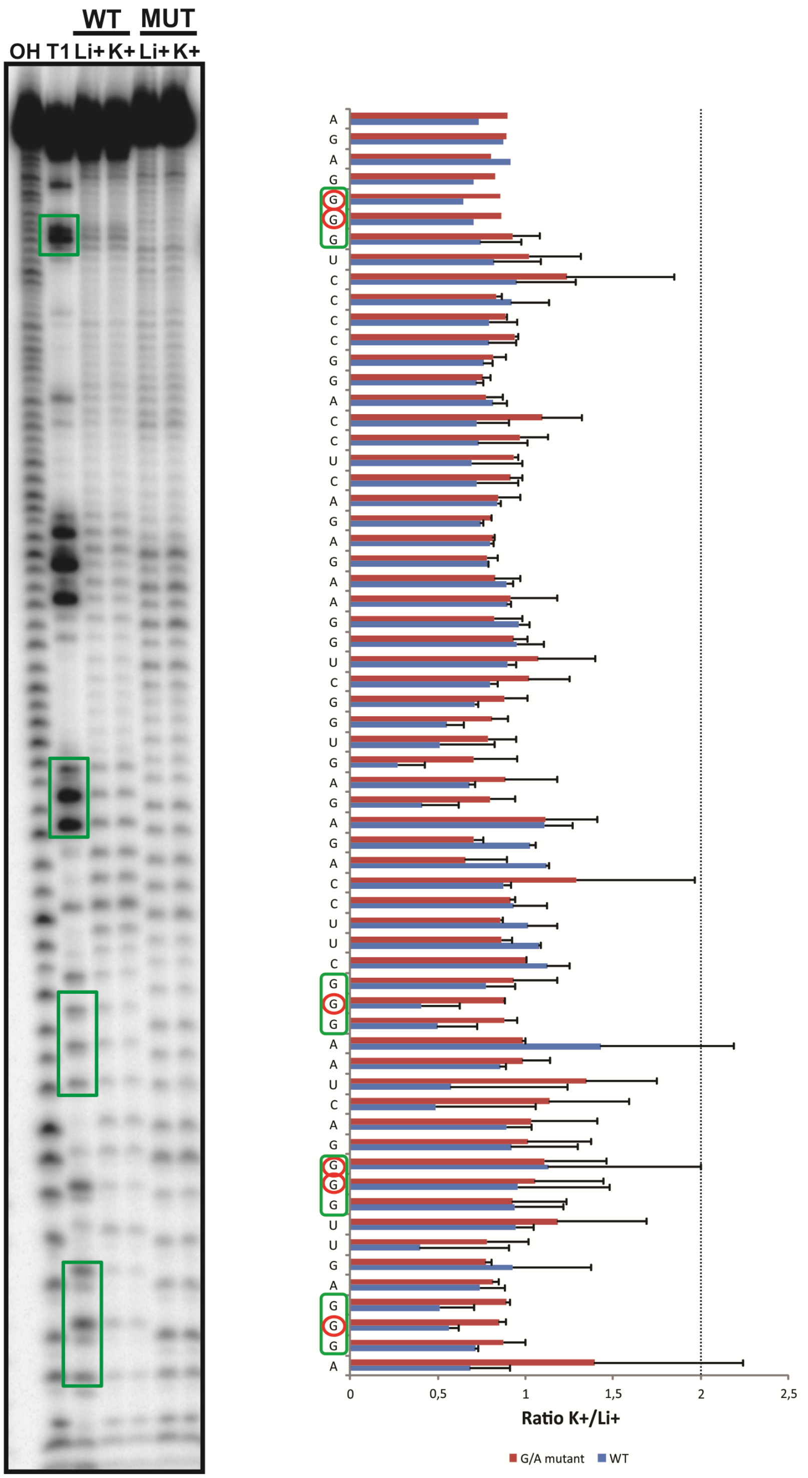

36 nt

GGUCUGCAGGGAGUUGGGGACUAAGGGCUUCCAGAGAGUGGCUGGAAGAGACUCCAGGCCCCUGGGGAGACUGUACUGU 2.39

PTPRU

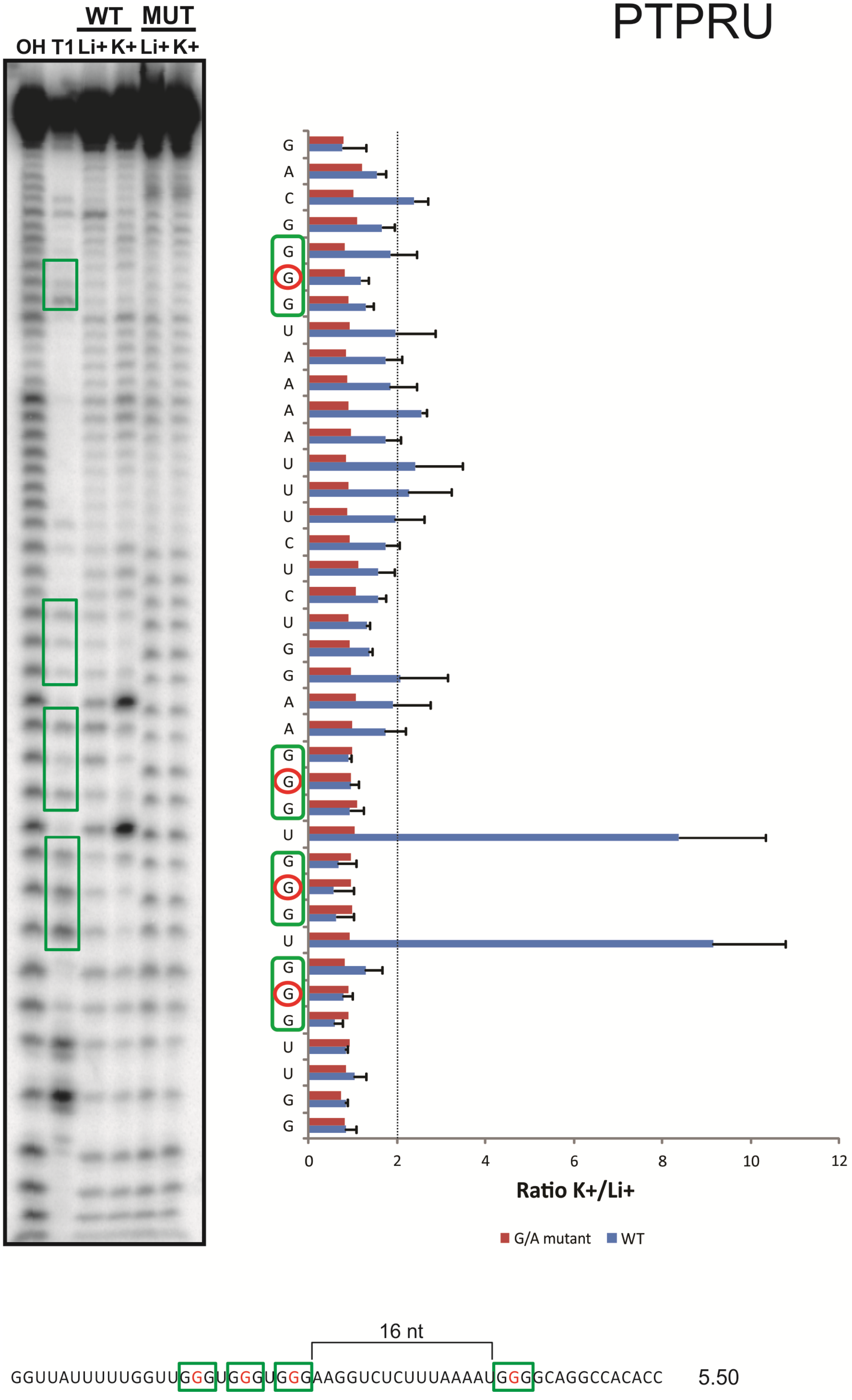

WT
MUT
OH T1 Li+ K+ Li+ K+
G
A
C
G
G
G
G
U
A
A
A
A
U
U
U
C
U
C
U
G
G
A
A
G
G
G
U
G
G
G
U
G
G
G
U
U
G
G
0
2
4
6
8
10
12
Ratio K+/Li+
G/A mutant
WT
16 nt
GGUUAUUUUUGGUUGGGUGGGUGGGAAGGUCUCUUUAAAAUGGGGCAGGCCACACC
5.50

RNF111

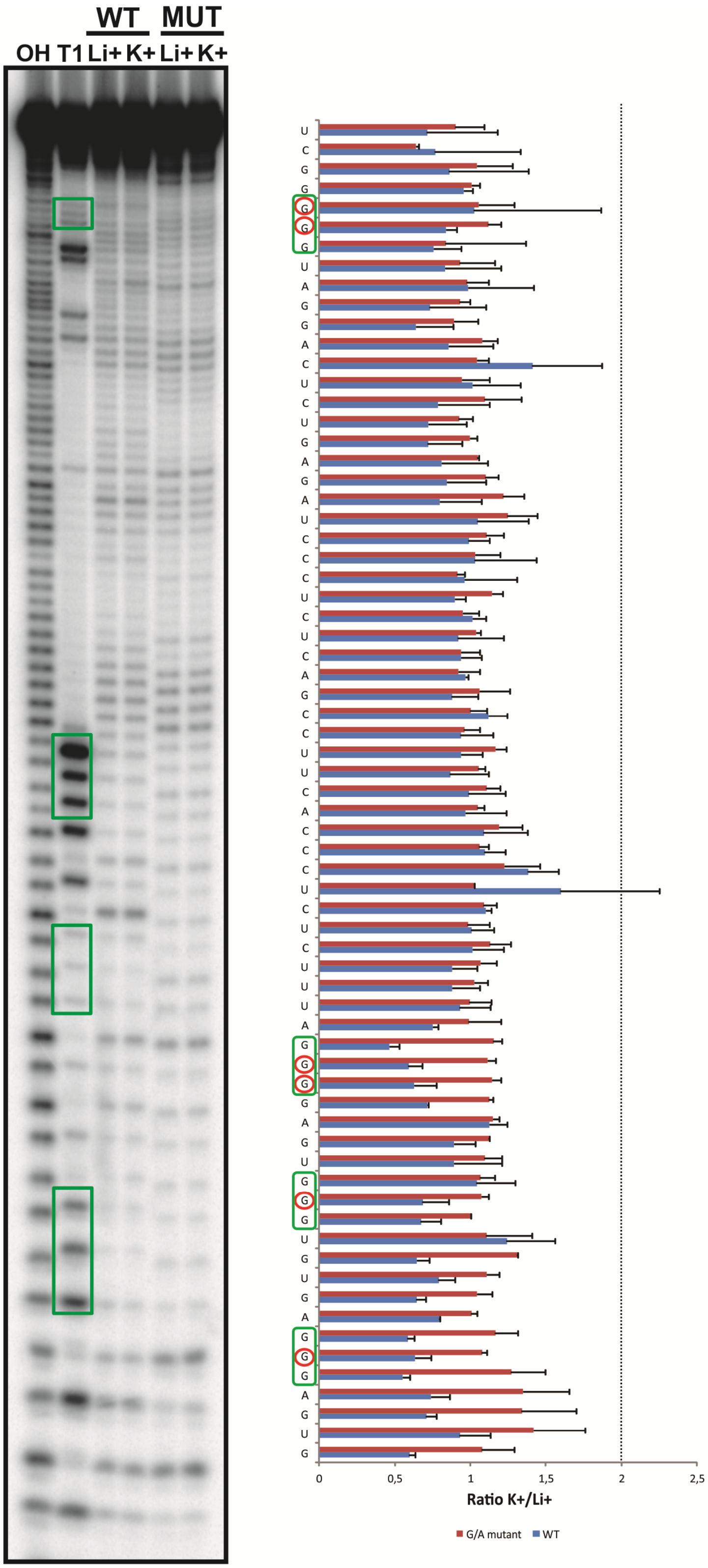

40 nt

GGCCUACAGUGAGGGAGUGUGGGUGAGGGGAUUUCUCUCCCACUUCCGACUCUCCCUAGAGUCUCAGGAUGGGGGCUGAGGAC 2.53

STRIP2

WT MUT
OH T1 Li+ K+ Li+ K+

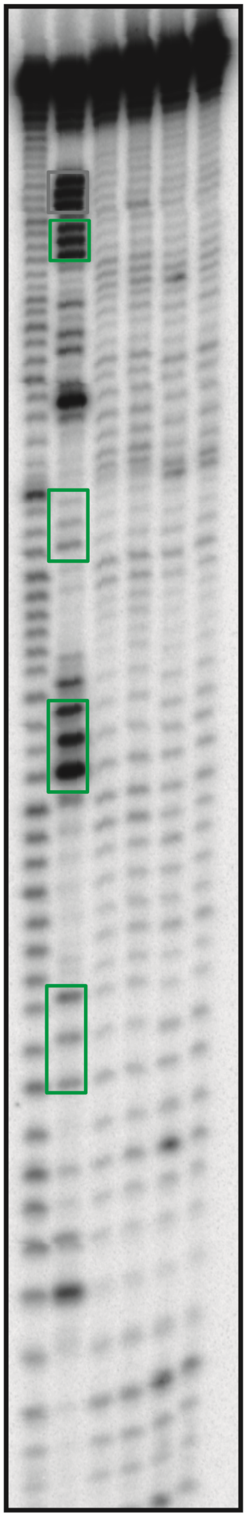

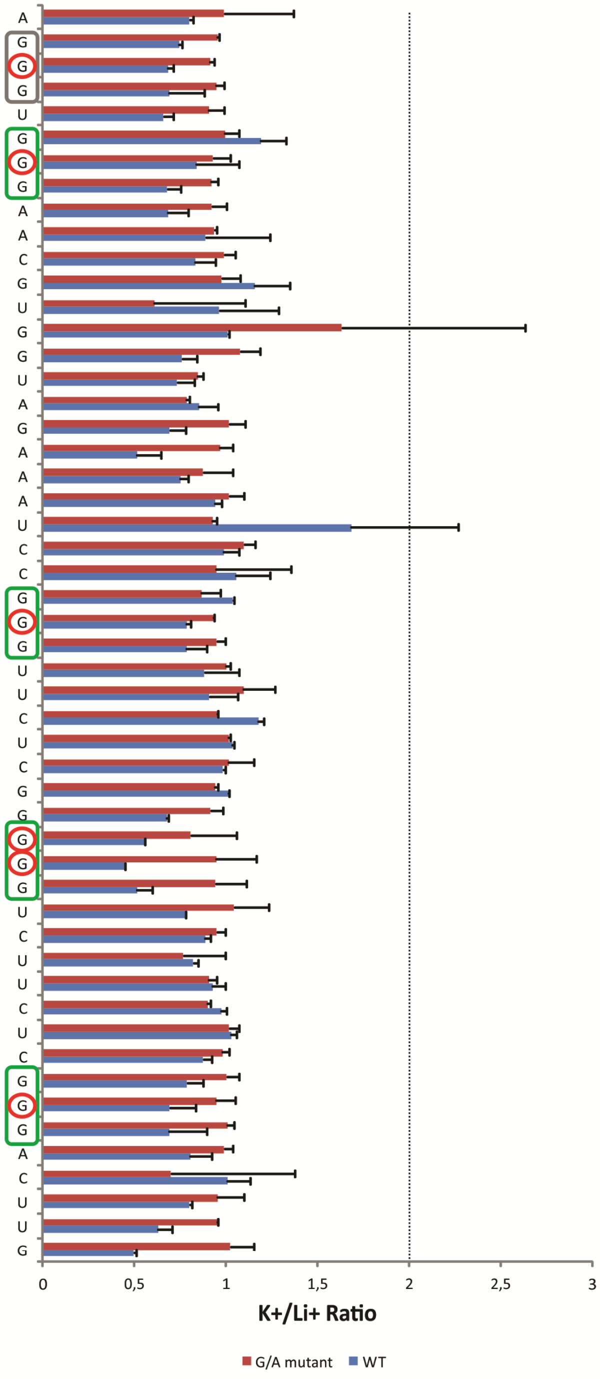

16 nt

GGCGCUGUUACCAGUUCAGGGCUCUUCUGGGGGCUCUUGGGCCUAAAGAUGGUGCAAGGGUGGGAUCCUGAAU 4.23

TNRC6C

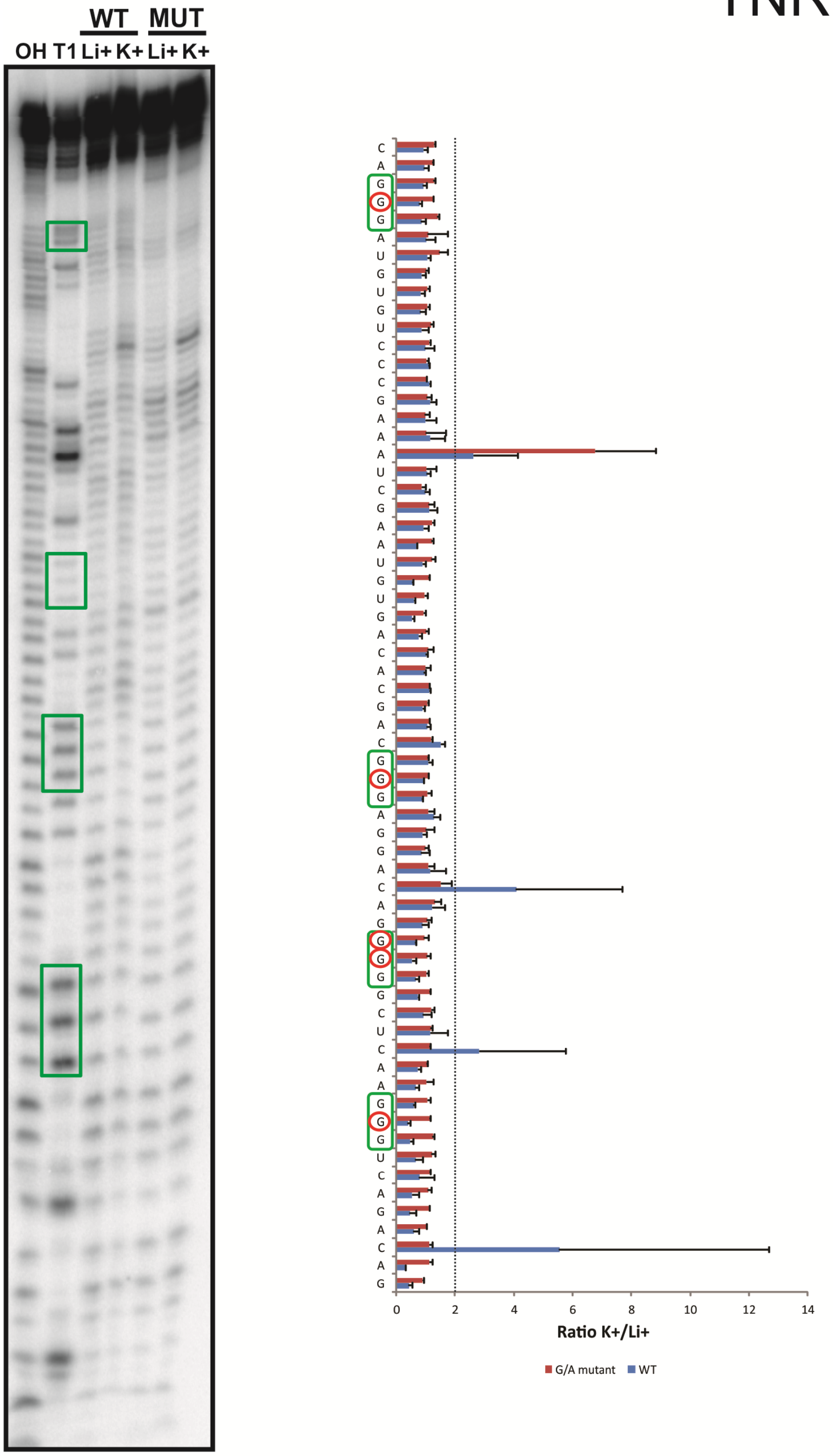

29 nt

GGAUUUUGACAGACUGGGAACUCGGGGACAGGAGGGCAGCACAGUGUAAGCUAAAGCCCUGUGUAGGGACCCCACGCAAAGA 2.05
