# Supplementary material for: Irregular G-quadruplexes Found in the Untranslated Regions of Human mRNAs Influence Translation
Source: J Biol Chem. 2016 Aug 24;291(41):21751–60. doi: 10.1074/jbc.M116.744839 (PMC5076843; doi:10.1074/jbc.M116.744839)
Supplement: Supplemental Data [file 10.1074_M116.744839_jbc.M116.744839-4.docx]

**Supplementary information**

Supplemental information S-4

Oligodeoxynucleotides used to generate DNA templates that have been transcribed for in-line probing experiments.

| Oligodeoxynucleotide | Sequence (5’ -> 3’) |
| --- | --- |
| BNIP1 WT | CGCACCACCTTCTTTCCCTCCCCACCCAGTCTCGGGGCAGCGCCCCCTAGAGCCtatagtgagtcgtatta |
| BNIP1 G/A mut | CGCACCACCTTCTTTCtCTCttCACtCAGTCTCGGGGCAGCGCtCtCTAGAGCCtatagtgagtcgtatta |
| DDX43 WT | CCCGCACGAGGCTAGCCCCCCCGCCCACGCTCTCTATCCCGCCCACTTCGCACTGCCtatagtgagtcgtatta |
| DDX43 G/A mut | CCCGCACGAGGCTAGCtCCCtCGCtCACGCTCTCTATCtCGCCCACTTCGCACTGCCtatagtgagtcgtatta |
| AVPR1B WT | CTCTAACTCCAACCCTTACCCTCCCAGCTCTCATTTCCAGTGCCCGAGGTGGGCAGAGAACCtatagtgagtcgtatta |
| AVPR1B G/A mut | CTCTAACTCCAACTCTTACTCTCTCAGCTCTCATTTCCAGTGCTCGAGGTGGGCAGAGAACCtatagtgagtcgtatta |
| B3GNT8 WT | GGATATGAATTTCCCCTCCTCCCTCCCATCAAGACCTGGAAGGAGGGGCTGAGCCAGGGGCCGGCCCCGGGGAACCtatagtgagtcgtatta |
| B3GNT8 G/A mut | GGATATGAATTTCTTCTCCTCTCTCTCATCAAGACCTGGAAGGAGGGGCTGAGCCAGGGGCCGGCTTCGGGGAACCtatagtgagtcgtatta |
| CYSRT1 WT | ACAGCCACCCCGCATGTCCCGGCATGTCCCGGCACTGTGTGAGCCACATTTGCTGAAGTCTGGGTCCTGGCCCTGGCAGGGCCtatagtgagtcgtatta |
| CYSRT1 G/A mut | ACAGCCACTTCGCATGTCTCGGCATGTCTCGGCACTGTGTGAGCCACATTTGCTGAAGTCTGGGTCCTGGCTCTGGCAGGGCCtatagtgagtcgtatta |
| DAG1 WT | CCTGGGTCCACCCACCCACCCTCAATCAGGGAATGGATACATTATTGCGAAAGGCATTCCTCCCTAAAAAGTACCtatagtgagtcgtatta |
| DAG1 G/A mut | CCTGGGTCCACTCACTCACTCTCAATCAGGGAATGGATACATTATTGCGAAAGGCATTCCTCTCTAAAAAGTACCtatagtgagtcgtatta |
| DUSP15 WT | CTTTAAGGGTGGGCCCCCTCCCCCAGCCCAAGGACTAAGGCACCAGGTGGCTGCAGCAGGCCGGCCCGGACACAGAGACGCCtatagtgagtcgtatta |
| DUSP15 G/A mut | CTTTAAGGGTGGGCCTTCTCTTCCAGCTCAAGGACTAAGGCACCAGGTGGCTGCAGCAGGCCGGCTCGGACACAGAGACGCCtatagtgagtcgtatta |
| KIF26A WT | CGCGTGGCCGGCCCTCCCTCCCCCACACTCCGTCTCCTCTCTGAGACTCCCGTCCTGCACAGAAACCtatagtgagtcgtatta |
| KIF26A G/A mut | CGCGTGGCCGGCTCTCTCTCCTTCACACTCCGTCTCCTCTCTGAGACTCTCGTCCTGCACAGAAACCtatagtgagtcgtatta |
| SPHK2 WT | GGATATGAATTTCCCCGCCCCCACCCGCGGGGAGAGGAACATTAGTGCAAATCCTAGCGCCGGCCCCGGGGAACCTGCCtatagtgagtcgtatta |
| SPHK2 G/A mut | GGATATGAATTTCCTCGCTTCCACTCGCGGGGAGAGGAACATTAGTGCAAATCCTAGCGCCGGCTTCGGGGAACCTGCCtatagtgagtcgtatta |
| TADA3 WT | GAAGTGGGCCTCCCTTCCCCTCCCCTAGGCCAGATAATCAGCCTGAGGCAGGGGTGAGGGCTACCCATCCAGCAGCTTCCtatagtgagtcgtatta |
| TADA3 G/A mut | GAAGTGGGCCTCTCTTCTTCTCCTCTAGGCCAGATAATCAGCCTGAGGCAGGGGTGAGGGCTACTCATCCAGCAGCTTCCtatagtgagtcgtatta |
| GRIA1 WT | GGCGAAAAAGAAGCCCAGGTCCAATCATAGATTTGGTGTTTCCCCCCCTTTCCCTTTATTCTTCTCGCCtatagtgagtcgtatta |
| GRIA1 G/A mut | GGCGAAAAAGAAGCTCAGGTCCAATCATAGATTTGGTGTTTCTCTCTCTTTCTCTTTATTCTTCTCGCCtatagtgagtcgtatta |
| RNF111 WT | GTCCTCAGCCCCCATCCTGAGACTCTAGGGAGAGTCGGAAGTGGGAGAGAAATCCCCTCACCCACACTCCCTCACTGTAGGCCtatagtgagtcgtatta |
| RNF111 G/A mut | GTCCTCAGCCTTCATCCTGAGACTCTAGGGAGAGTCGGAAGTGGGAGAGAAATCTTCTCACTCACACTCTCTCACTGTAGGCCtatagtgagtcgtatta |
| TEF WT | GCGTCGGACATCGTGCCCCGGACTCGGCTCGCGCACCTCGCCTCCCCCGCCCGCCCCCCGAGATGGGCCtatagtgagtcgtatta |
| TEF G/A mut | GCGTCGGACATCGTGCTTCGGACTCGGCTCGCGCACCTCGCCTCTCTCGCTCGCTTCTCGAGATGGGCCtatagtgagtcgtatta |
| DCTN5 WT | GGCAAGTCCTAAATCCCAGACTCATTTGTTCACTTGCTTGCTTCCTCCCTCCCTCCCTACCAAAACACAGTATGATCCtatagtgagtcgtatta |
| DCTN5 G/A mut | GGCAAGTCCTAAATCTCAGACTCATTTGTTCACTTGCTTGCTTCCTCTCTCTCTCTCTACCAAAACACAGTATGATCCtatagtgagtcgtatta |
| DOK1 WT | ATCCCTCTGGTCCCCTGGTCTCACACAGCATGGCCCCTCCCACCCTCTGGCTTCTGCCtatagtgagtcgtatta |
| DOK1 G/A mut | ATCCCTCTGGTCCTCTGGTCTCACACAGCATGGCTTCTCTCACTCTCTGGCTTCTGCCtatagtgagtcgtatta |
| MTF1 WT | TTCTGCAAACTTGTCCCTCTGGACACATTCAGCATTATACAAAGTCTCTCAGGAAAACCCACCCACCCTCCAATATCCCtatagtgagtcgtatta |
| MTF1 G/A mut | TTCTGCAAACTTGTCTCTCTGGACACATTCAGCATTATACAAAGTCTCTCAGGAAAACTCACTCACTCTCCAATATCCCtatagtgagtcgtatta |
| PLXNB1 WT | ACAGTACAGTCTCCCCAGGGGCCTGGAGTCTCTTCCAGCCACTCTCTGGAAGCCCTTAGTCCCCAACTCCCTGCAGACCtatagtgagtcgtatta |
| PLXNB1 G/A mut | ACAGTACAGTCTCCTCAGGGGCCTGGAGTCTCTTCCAGCCACTCTCTGGAAGCTCTTAGTCTTCAACTCTCTGCAGACCtatagtgagtcgtatta |
| PTPRU WT | GGTGTGGCCTGCCCCATTTTAAAGAGACCTTCCCACCCACCCAACCAAAAATAACCtatagtgagtcgtatta |
| PTPRU G/A mut | GGTGTGGCCTGCCTCATTTTAAAGAGACCTTCTCACTCACTCAACCAAAAATAACCtatagtgagtcgtatta |
| STRIP2 WT | ATTCAGGATCCCACCCTTGCACCATCTTTAGGCCCAAGAGCCCCCAGAAGAGCCCTGAACTGGTAACAGCGCCtatagtgagtcgtatta |
| STRIP2 G/A mut | ATTCAGGATCTCACTCTTGCACCATCTTTAGGCTCAAGAGCCTTCAGAAGAGCTCTGAACTGGTAACAGCGCCtatagtgagtcgtatta |
| TNRC6C WT | TCTTTGCGTGGGGTCCCTACACAGGGCTTTAGCTTACACTGTGCTGCCCTCCTGTCCCCCGAGTTCCCAGTCTGTCAAAATCCtatagtgagtcgtatta |
| TNRC6C G/A mut | TCTTTGCGTGGGGTCTCTACACAGGGCTTTAGCTTACACTGTGCTGCTCTCCTGTCCTTCGAGTTCTCAGTCTGTCAAAATCCtatagtgagtcgtatta |

Lower case = T7 promoter

Supplemental information S-5

Oligodeoxynucleotides used to PCR amplify and clone full length 5’ and 3’ UTR into psiCHECK2.1 vector for luciferase assays.

| Oligodeoxynucleotides | Sequence (5’ -> 3’) |
| --- | --- |
| DDX43 F_speI | CAGTCAGTACTAGTAGGGAGCAAATTGGGCGTTTG |
| DDX43 R_sacI | CAGTCAGTGAGCTCTGTTCCAAGAAGGGGCGCGTC |
| AVPR1B F_pmeI | CAGTCAGTGTTTAAACGAAAGACTCGCTGGGGTCTGGTAC |
| AVPR1B R_notI | CAGTCAGTGCGGCCGCCTGCCACCCGTGGACCCCTAGAC |
| KIF26A F_pmeI | CAGTCAGTGTTTAAACGGCTGGGCGCCGGACAAGAG |
| KIF26A R_notI | CAGTCAGTGCGGCCGCCATCGATGCATCTTTAATTCAC |
| BNIP1 5_UTR For speI | CAGTCAGTACTAGTCTCTAGGGGGCGCTGCCCCGAGACTGGGTGGGGAGGGAAAGAAGGTGGT |
| BNIP1 5_UTR Rev sacI | CAGTCAGTGAGCTCGTTGGGGACGCCGGCTACGGGCAGCGGCAGGACCCAAGTTCAAACGCGTTTTCGCACCACCTTCTTTCCCTCCCCACCCAGTC |
| BNIP1 G/A mut 5_UTR For speI | CAGTCAGTACTAGTCTCTAGAGAGCGCTGCCCCGAGACTGAGTGAAGAGAGAAAGAAGGTGGT |
| BNIP1 G/A mut 5_UTR Rev sacI | CAGTCAGTGAGCTCGTTGGGGACGCCGGCTACGGGCAGCGGCAGGACCCAAGTTCAAACGCGTTTTCGCACCACCTTCTTTCTCTCTTCACTCAGTC |
| GRIA1 F_speI | CAGTCAGTACTAGTATAGAGCTTGCTGCCTGTG |
| GRIA1 R_sacI | CAGTCAGTGAGCTCATTCCTTTTTGCATTGGCG |
| DOK1 F_xhoI | CAGTCAGTCTCGAGGAAGGACGGCAAGGCTGAGGTGG |
| DOK1 R_notI | CAGTCAGTGCGGCCGCTTCTTAAACCCAAGCTGACTTTAATG |
| PTPRU F_xhoI | CAGTCAGTCTCGAGCGGGGCCCTGGCCTGGGGC |
| PTPRU R_notI | CAGTCAGTGCGGCCGCCAGGGCTGCTCTGTGGCATTCAGAG |
| TEF 5_UTR For speI | CAGTCAGTACTAGTATTGGGCGCCTGCGCAGTAGCTGCCCGTGTCGGCAGCTGCAGCGGGTCGCACGGCTCCGGCCCATCTCGGGGGGCGGGCG |
| TEF 5_UTR Rev sacI | CAGTCAGTGAGCTCCGTGCCCCGGACTCGGCTCGCGCACCTCGCCTCCCCCGCCCGCCCCCCGAGATGGGCCGGAG |
| TEF GA mut 5_UTR For speI | CAGTCAGTACTAGTATTGGGCGCCTGCGCAGTAGCTGCCCGTGTCGGCAGCTGCAGCGGGTCGCACGGCTCCGGCCCATCTCGAGAAGCGAGCG |
| TEF GA mut 5_UTR Rev sacI | CAGTCAGTGAGCTCCGTGCTTCGGACTCGGCTCGCGCACCTCGCCTCTCTCGCTCGCTTCTCGAGATGGGCCGGAG |

Supplemental information S-6

Complete 5’UTR and 3’UTR cloned upstream and downstream of the Renilla luciferase gene. The G4 forming sequences are in highlighted gray.

Homo sapiens arginine vasopressin receptor 1B (AVPR1B) 3’UTR NM_000707.3 (340 nt)

gaaagactcgctggggtctggtactgcccccaggactagtggaggttctctgcccacctcgggcactggaaatgagagctgggagggtaagggttggagttagaggagccctgtctaaagcggagcgaaaaggccagaatgggtcccctaccctggtgtcacagctgcccctagtgtgagggctgcctcataagctcccaatctcagacactggcagtcagggagaatcaaactgcctgtctccctggtcctgccatattcatagggtgtccatgcacacatggtgtcccagatctaggcaggcctaggatggtgctgtctaggggtccacgggtggcag

Homo sapiens BCL2/adenovirus E1B 19kDa interacting protein 1 (BNIP1) 5’UTR NM_013978.2 (104 nt)

ctctagggggcgctgccccgagactgggtggggagggaaagaaggtggtgcgaaaacgcgtttgaacttgggtcctgccgctgcccgtagccggcgtccccaac

Homo sapiens DEAD (Asp-Glu-Ala-Asp) box polypeptide 43 (DDX43)5’UTR NM_018665.2 (344 nt)

agggagcaaattgggcgtttggaaaaagtcccacgcctggaaaggaatacacagtgggggggtggggggcggggcacatcgactacttcaaggcacactcccacggttcagaaaaccgattggctaaaactgggaaagtcccgcccacaacgctcactggccaatggctgcgtgaaatcagtgcgaagtgggcgggatagagagcgtgggcgggggggctagcctcgtgcgggctccttaagtagcggctgcgtggcttccctggcacgctactcttacgacgtcacggtcaggtggtgcagagctggacggcaacgacgtcggacgcgccccttcttggaacaATG

Homo sapiens kinesin family member 26A (KIF26A) 3’UTR NM_015656.1 (1101 nt)

ggctgggcgccggacaagaggagggggcgtgcagcgggctggaggacgggacgtgggacggagcgaggatgtggtgggggctgcggggggaggatgcggaggggtttctgtgcaggacgggagtctcagagaggagacggagtgtgggggagggagggccggccacgcggtggacagagcgagggtgccagggtgaccagaagaccgtcaccacccgacagcaacgcaagtgcctttgaccttgatttggacttttctcccttttgcatttggtgctacagacttgagacaccagcagaagttgtgttcagcccggccccgctgcgcctgtccgggccggggctggcgccggttgtgtttgtgtccaccttgccttctttgcagccaagcagtttttgtggagtggagtgggacttacctgcacgccccaggggtctttcaggattcaggatgacttttcttttacaatggtttcctctcggcagagcccgggttgtgggggatctgtgtggggttctcaacgcagatccatcctggggtctcccgggcagggatggctgacctcgagtcccctcccttcccgagaacccgctctgtcccgagggcagctaacaagggctgagccccaggtacaggttgcctcttccacggcaggaatttttaccaaaaccacaagcaaaaaacaaaacagaccaccacgaccaacaacaaagatggggggtagggttttgtaaaggttctgttaggttcatatttttatatcattttgcccataaatgcggaatttgccgtgggaatttgaagacaaatgatctatgtttttatggttttctagggaaggtgttctgggggccgggctctctccagctgtgggaggcctgctccctctggggggcaccctgggcagggtgggggggccttgggaggcgcttcttgccaaatgcagacgaggggtgagcctgccagcgtttgcgacgtccccgcacgacaggctcatactttctgaggatcgtgcatagcataggacgtctgaacctttgtacaaatgtgtagatgacatcttgctacagcttttatttgtgaattaaagatgcatcgatg

Homo sapiens docking protein 1, 62kDa (downstream of tyrosine kinase 1) (DOK1) 3’UTR NM_001197260.1 (437 nt)

gaaggacggcaaggctgaggtggctaagggggaccatggggaggtggcactagggatcaaagaagatggttagaaccagcagaagccagagggtgggaggggccatgctgtgtgagaccaggggaccagagggatgggagagtcaagggaaggacaatcccaggaagtcctaagaagtggggcagatggcagggctgaggatgggctctgcatcccccaaagccatcccttccctacttccccaaatgaagggacggctgtgggaccaggtctgtggaaagtggtgcatggtcagaatgggtgcagtttgaggggcctgtgtggaggcctcagggagatgttggactgtgcctggatccttactcctgcattgttctttgccagagacctatttaaaaattttaaaattctcattaaagtcagcttgggtttaagaa

Homo sapiens glutamate receptor, ionotropic, AMPA 1 (GRIA1) 5’UTR NM_000827.3 (365 nt)

atagagcttgctgcctgtgtgagtgtgagggggagagcgagagagagcaagggagggagagagaggcaggctgcgaggggagaggagagggagtgggggagccagcgctccagctagcatgaggacgggcttcttttcccgtgctcagttaatctggctgtcagttggtgttaacgctgcagtttaagtgttcggattccaagggaaacagacaaacctcacgaaaggaaggaagcaagcaagcaaggaaggaactgcaggaggaaaagaacaggcagaacagcgagaagaataaagggaaagggggggaaacaccaaatctatgattggacctgggcttctttttcgccaatgcaaaaaggaat

Homo sapiens protein tyrosine phosphatase, receptor type, U (PTPRU) 3’UTR NM_133178.3 (1152 nt)

cggggccctggcctggggcacccactgcacactcagggccagacccaccatcctggactggcgaggaagatcagtgcctcctgctctgcccaaacacactcccatggggcaagcactggagtggatgctgggctatcttgctcccccttccactgtgggcagggcctttcgcttgtcccatgggcgggtggtgggccaaggaggagcttagcaagtctgcagcccagccccacctccatagggtcctgcaggcctgtgctgagaggcctggtgctgcctggcagagtgacaaaggctcaggacggctggctctgggggactcaggccaagccccttggcaccatcctggcttttggcagggatgagtgaggccctgcagagagcatcccaggccaaggttcccactcagcctgccccctctgcatgtgggtagaggatgtactgggacttggcatttaggattccatctggcccagcccctgaaggtcctggggaagcaggtctcaattctgaatagccagtggggcacactgactgtcctccccaggggaactgcagcgccctcctccccactgccccctgcagcccctgagatattttgctcactatccctccccacttgcttccctgatatgtgctctgagcttccctgaaccaggatctgcctattactgctgtgccccatggggggctccttccctgcctgacccactgttgcagaatgaagtcacctcgcccccctcttcctttaatcttcaggcctcactggcctgtcctgctcagcttgggccagtgacaatctgcaaggctgaacaacagcccctggggttgaggcccctgtggctcctggtcaggctgcccgttgtggggaggggcagtgttagagcagggctggtcataccctctggagttcagaggaagaggtaggaccagtgcttttttgtttcttttgttatttttggttgggtgggtgggaaggtctctttaaaatggggcaggccacacccccattccgtgcctcaatttccccatctgtaaactgtagatatgactactgacctacctcgcagggggctgtggggaggcataagctgatgtttgtaaagcgctttgtaaataaacgtgctctctgaatgccacagagcagccctg

Homo sapiens thyrotrophic embryonic factor (TEF) 5’UTR NM_003216.3 (116 nt)

attgggcgcctgcgcagtagctgcccgtgtcggcagctgcagcgggtcgcacggctccggcccatctcggggggcgggcgggggaggcgaggtgcgcgagccgagtccggggcacg
